# Cytoplasmic TDP43 Binds microRNAs: New Disease Targets in Amyotrophic Lateral Sclerosis

**DOI:** 10.3389/fncel.2020.00117

**Published:** 2020-05-12

**Authors:** Ximena Paez-Colasante, Claudia Figueroa-Romero, Amy E. Rumora, Junguk Hur, Faye E. Mendelson, John M. Hayes, Carey Backus, Ghislaine F. Taubman, Laurie Heinicke, Nils G. Walter, Sami J. Barmada, Stacey A. Sakowski, Eva L. Feldman

**Affiliations:** ^1^Department of Neurology, University of Michigan, Ann Arbor, MI, United States; ^2^Department of Biomedical Sciences, School of Medicine and Health Sciences, University of North Dakota, Grand Forks, ND, United States; ^3^Single Molecule Analysis Group, Department of Chemistry, University of Michigan, Ann Arbor, MI, United States

**Keywords:** amyotrophic lateral sclerosis, trans-activation response element DNA/RNA-binding protein of 43 kDa (TDP43), microRNAs, cytoplasmic aggregates, profiling

## Abstract

Amyotrophic lateral sclerosis (ALS) is a progressive, fatal, and incurable neurodegenerative disease. Recent studies suggest that dysregulation of gene expression by microRNAs (miRNAs) may play an important role in ALS pathogenesis. The reversible nature of this dysregulation makes miRNAs attractive pharmacological targets and a potential therapeutic avenue. Under physiological conditions, miRNA biogenesis, which begins in the nucleus and includes further maturation in the cytoplasm, involves trans-activation response element DNA/RNA-binding protein of 43 kDa (TDP43). However, TDP43 mutations or stress trigger TDP43 mislocalization and inclusion formation, a hallmark of most ALS cases, that may lead to aberrant protein/miRNA interactions in the cytoplasm. Herein, we demonstrated that TDP43 exhibits differential binding affinity for select miRNAs, which prompted us to profile miRNAs that preferentially bind cytoplasmic TDP43. Using cellular models expressing TDP43 variants and miRNA profiling analyses, we identified differential levels of 65 cytoplasmic TDP43-associated miRNAs. Of these, approximately 30% exhibited levels that differed by more than 3-fold in the cytoplasmic TDP43 models relative to our control model. The hits included both novel miRNAs and miRNAs previously associated with ALS that potentially regulate several predicted genes and pathways that may be important for pathogenesis. Accordingly, these findings highlight specific miRNAs that may shed light on relevant disease pathways and could represent potential biomarkers and reversible treatment targets for ALS.

## Introduction

Amyotrophic lateral sclerosis (ALS) is a neurodegenerative disease characterized by cortical, brainstem, and spinal cord motor neuron loss that results in progressive skeletal muscle weakness and atrophy (Brown and Al-Chalabi, 2017). There is currently no effective cure and most patients die within 3–5 years of diagnosis. The disease can occur as familial ALS (fALS), which constitutes around 15% of ALS incidence, or the more frequent sporadic ALS (sALS; Brown and Al-Chalabi, 2017; Chia et al., 2018; Goutman et al., 2018; Oskarsson et al., 2018).

The precise underlying etiology of ALS is not completely understood; however, a genetic cause has been identified in approximately 70% of fALS cases (Chia et al., 2018). The associated genetic changes (Brown and Al-Chalabi, 2017; Chia et al., 2018; Goutman et al., 2018) include expansions in C9orf72 (Balendra and Isaacs, 2018) and mutations in the RNA-binding proteins trans-activation response element DNA/RNA-binding protein of 43 kDa (TDP43; Sreedharan et al., 2008) and fused in sarcoma (FUS; Kwiatkowski et al., 2009; Vance et al., 2009). fALS and sALS share similarities in histopathological features, with over 90% of cases exhibiting TDP43 protein inclusions in the cytoplasm of the diseased brain and spinal cord neurons and glia (Hardiman et al., 2017). Moreover, aberrant protein and RNA metabolism and altered epigenetic mechanisms, such as those involving microRNAs (miRNAs), are recurrent themes among the dysregulated pathways (Vucic et al., 2014; Paez-Colasante et al., 2015; Brown and Al-Chalabi, 2017; Jimenez-Pacheco et al., 2017).

miRNAs are evolutionarily conserved ~22 nucleotide-long non-coding RNAs that post-transcriptionally regulate several mRNA targets, generally by binding to their 3′ untranslated region, which marks them for translational repression and eventual degradation (Lagos-Quintana et al., 2001; Lau et al., 2001; Lee and Ambros, 2001). This regulation alters the gene expression profile and elicits changes downstream, thereby modulating physiological processes (Engels and Hutvagner, 2006; Selbach et al., 2008). miRNAs undergo a complex maturation process that includes the interaction of its primary (pri-miRNA) and precursor forms (pre-miRNA) with nuclear and cytoplasmic enzyme complexes and RNA binding proteins, including TDP43 (Kawahara and Mieda-Sato, 2012; Loffreda et al., 2015). miRNAs are highly expressed in the nervous system, and miRNA dysregulation occurs in multiple neurodegenerative diseases (Paez-Colasante et al., 2015; Tan et al., 2015; Maoz et al., 2017; Quinlan et al., 2017; Singh and Sen, 2017; Dolati et al., 2018), including ALS (Paez-Colasante et al., 2015; Rinchetti et al., 2018; Gagliardi et al., 2019). Of particular relevance, differentially expressed miRNAs have been detected in ALS patient spinal cord autopsy tissue, cerebrospinal fluid (CSF), and plasma, and are proposed as potential ALS biomarkers (Campos-Melo et al., 2013; Takahashi et al., 2015; Figueroa-Romero et al., 2016; Joilin et al., 2019). The driving force for miRNA dysregulation in ALS, however, has not been fully elucidated.

Complex molecular networks and dynamic cross-talk are likely involved in ALS-associated dysregulation (Jobe et al., 2012; Poddar et al., 2017), and TDP43, as an RNA binding protein, may play an integral role (Birsa et al., 2020). Over 50 mutations to *TARDBP*, the gene encoding TDP43, occur in fALS (Kapeli et al., 2017). While TDP43 normally shuttles between the nucleus and cytoplasm (Chen-Plotkin et al., 2010; Bhardwaj et al., 2013; Gao et al., 2018), mutations and cellular stress skew the localization of mutant and/or wild-type (WT) TDP43 from the nucleus to the cytoplasm (Barmada et al., 2010), generating cytoplasmic TDP43 (cyTDP43) inclusions (Neumann et al., 2006; Zhang et al., 2008; Liu-Yesucevitz et al., 2010). WT or mutant TDP43 overexpression in motor neuron-like cells also interferes with miRNA biogenesis (Emde et al., 2015), and overexpressed mutant TDP43 promotes the formation of stress granules, which interact with and inhibit miRNA processing (Emde et al., 2015; Paez-Colasante et al., 2015; Weskamp and Barmada, 2018; Chen and Cohen, 2019). Additionally, TDP43 knockdown in neuroblastoma cells markedly affects miRNA levels (Kawahara and Mieda-Sato, 2012; Di Carlo et al., 2013), and TDP43 profoundly influences vital cellular processes through binding to several RNA targets in neurons (Sephton et al., 2011; Honda et al., 2013). Taken together, these findings are consistent with a role for TDP43 inclusions in RNA dysregulation during ALS and constitute a potential pathological mechanism that may lead to neuronal injury.

While suggestive of a causal relationship, however, our current understanding lacks insight into specific mechanisms whereby cyTDP43 inclusions may interfere with miRNA bioavailability and/or function. To address this gap in our knowledge, we used a high-throughput assessment of miRNA levels associated with multiple TDP43 pathological states to identify specific changes in miRNA/protein binding linked with cyTDP43. Our results expand the understanding of how TDP43 mislocalization affects specific miRNA levels in ALS, and thereby provide a foundation to ultimately gain new mechanistic insights and uncover untapped diagnostic and therapeutic avenues in ALS.

## Materials and Methods

### TDP43-miRNA Binding Assays

We used a native gel mobility shift assay (Ramsey and Kelm, 2009; Poddar et al., 2017) to assess the formation of TDP43-miRNA complexes for three miRNAs known to associate with TDP43: miR-132, miR-143, and miR-574-5p (Buratti et al., 2010; Kawahara and Mieda-Sato, 2012; Freischmidt et al., 2013). The select miRNAs were purchased from Integrated DNA Technologies (Coralville, IA, USA), 5′end-labeled with ^32^P using T4 polynucleotide kinase (PNK; New England Biolabs, Ipswitch, MA, USA), and excess unincorporated isotope was removed with a Centri-Spin 10 column (Invitrogen, Thermo Fisher Scientific, Carlsbad, CA, USA). Binding affinity was measured by mixing the 5′-^32^P-end labeled miRNAs with excess denatured full-length TDP43 (0.05, 0.125, 0.25, 0.5, or 1.25 μM; cat# ab156345, Abcam, San Francisco, CA, USA) for 30 min at room temperature in buffer [14 mM Tris (pH 8.0), 35 mM KCl, 14 mM NaCl, 1.4 mM MgCl_2_, 14% glycerol, 0.7 mM dithiothreitol (DTT)]. Samples were then electrophoresed for 3 h at 4°C on a native 0.5× Tris-borate-EDTA (TBE) gel (19:1 crosslink). Bands were visualized by exposure to a storage PhosphorImager screen for 30 min and scanned by a Typhoon PhosphorImager (GE Healthcare, Chicago, IL, USA).

Apparent binding affinities of TDP43 to select miRNAs of varying UG repeats (bolded and underlined below) were next measured *via* a direct colorimetric enzyme-linked immunosorbent assay (ELISA; Rumora et al., 2010, 2013). Briefly, 3′-biotinylated miRNAs (miR-574-5p, 5′-**UG**AG**UGUGUGUGUGUG**AG**UGUG**U-3′; miR-652-3p, 5′-AA**UG**GCGCCACUAGGGU**UGUG**-3′; and miR-204-5p, 5′-UUCCCUU**UG**UCAUCCUA**UG**CCU-3′) were synthesized by Integrated DNA Technologies. Serial dilutions of each 3′-biotinylated miRNA were immobilized on a 96-well StreptaWell microplate (Roche Applied Science, Penzberg, Germany) at concentrations ranging from 0 to 20 nM for miR-574, 0–85 nM for miR-652, and 0–80 nM for miR-204 for 2 h at room temperature. Different concentration ranges were used for each 3′-biotinylated miRNA to achieve the saturable binding of TDP43 to individual miRNAs. Microplate wells were blocked with 2% ELISA grade BSA for 1 h at room temperature, and wells were washed after each step with wash buffer (20 mM HEPES pH 7.5, 150 mM NaCl, 1.5 mM MgCl_2_, 0.05% v/v Tween 20). For TDP43 binding to immobilized miRNAs, pure full length TDP43 protein (5 nM; cat# ab224788, Abcam) was incubated for 12 h overnight at 4°C in binding buffer (wash buffer supplemented with 0.5 mM DTT, 1.0 μg/ml of an AC/TG nonspecific oligonucleotide, 0.2% w/v ELISA grade BSA). After the 12 h incubation, immobilized miRNA-protein complexes were detected with purified anti-TDP43 mouse monoclonal antibody (1.0 μg/ml; R&D, Minneapolis, MN) in antibody buffer (wash buffer supplemented with 0.2% w/v ELISA grade BSA) for 1 h at room temperature. A 1:8,000 dilution of secondary horseradish peroxidase (HRP)-coupled goat anti-mouse antibody (Santa Cruz Biotechnology, Dallas, TX, USA) was then incubated for 1 h at room temperature. Finally, 100 μl of ABTS chromogenic substrate solution (Millipore, Burlington, MA, USA) was added to each well and incubated for 1 h. Colorimetric measurements were taken at A405 every 30 min until saturation (maximum A_405_ ~1) on a Synergy HTX multimode plate reader (BioTek, Winooski, VT, USA) equipped with Gen5 software (version 3.03). The EC50 values for each TDP43-miRNA apparent affinity were determined by fitting the datasets to a four-parameter variable slope equation in Prism 6 (GraphPad, San Diego, CA, USA).

### Plasmids

To generate stable doxycycline-inducible cell lines expressing eGFP-His-tagged TDP43 variants that primarily localized to the nucleus or cytoplasm, we first amplified eGFP-His from pGW1-T202-TDP43-eGFP-His (Barmada et al., 2010) using the primers 5′-ATA AGA ATG C**GG CC**G CAA CTA GAG CTG TTT GGG ACG-3′ and 5′-CGG A**CG CG**T TTT TAG TGA TGG TGA TGG TGA TG-3′. The eGFP-His fragment was subcloned directionally into the NotI-MluI restriction sites (underlined in bold in the primer sequence) of the doxycycline-inducible pLVX-TRE3G lentiviral vector (Clontech, Takara Bio USA, Mountain View, CA, USA) to generate the pLVX-TRE3G-**eGFP-His** construct. WT TDP43 from pGW1-TDP43WT-eGFP (Barmada et al., 2014) was next amplified using the primers 5′-CGG **GAT C**CA TGT CTG AAT ATA TTC GGG TAA CCG-3′ and 5′-ATA GTT TAG C**GG CC**G CCA TTC CCC AGC CAG AAG A-3′, and the WT TDP43 fragment was ligated directionally into the BamHI-NotI restriction sites (underlined in bold in the primer sequence) of the pLVX-TRE3G-eGFP-His construct to create the final pLVX-TRE3G-**WT-TDP43-eGFP-His** plasmid. PCR reactions were performed with Q5 High-Fidelity DNA Polymerase (New England BioLabs) and transformations with Stellar competent cells (Clontech, Takara Bio USA Mountain View, CA, USA), both according to the manufacturer’s instructions. Missense mutations disrupting the two nuclear localization signals (ΔNLS) in TDP43 to force cytoplasmic localization were next introduced into the pLVX-TRE3G-TDP43WT-eGFP-His plasmid using site-directed mutagenesis as previously described (Winton et al., 2008; Barmada et al., 2010) to generate pLVX-TRE3G-**ΔNLS-TDP43-eGFP-His**. All sequences were verified by the University of Michigan Sequencing Core^1^.

### Generation of Doxycycline-Inducible Stable HeLa Cell Lines

HeLa cells were grown in Dulbecco’s modified Eagle’s medium (DMEM; 11965-092, Gibco, Thermo Fisher Scientific, Gaithersburg, MD, USA) supplemented with 10% fetal bovine serum (A3160401, Thermo Fisher Scientific, Gaithersburg, MD, USA) at 37°C in 10% CO_2_. Confluent cells were transiently transfected with 100 ng of the pLVX-TRE3G-eGFP-His, pLVX-TRE3G-WT-TDP43-eGFP-His, or pLVX-TRE3G-ΔNLS-TDP43-eGFP-His constructs along with pLVX-EF1α-Tet3G (Clontech, Takara Bio USA) using Lipofectamine 2000 reagent (Invitrogen) according to the manufacturer’s protocol. Following a 6 h incubation, wells were rinsed and replenished with growth media containing the selection antibiotics puromycin (1 μg/ml; #P8833, Sigma, St. Louis, MO, USA) and geneticin (1 μg/ml; G418; #10131-035, Gibco) for 48 h. Doxycycline (1 μg/ml; #PHR1145, Sigma) was then added to the media to induce protein expression and confirm transfection. Transfection efficiency was determined to be about 20%, and stable lines were generated using a serial dilution protocol in 96-well plates (Corning Inc., Corning, NY, USA; Ryan, 2008). HeLa cells were monitored daily and scored for wells that contained a single colony, which was grown to confluence. Induction with doxycycline was performed to select eGFP-positive clones for expansion. A confluent sample from each expanded eGFP-expressing clone was fixed with 4% paraformaldehyde and stained with Hoechst reagent to visualize the nuclei and localize eGFP (*i.e*., nuclear vs. cytoplasmic). Multiple clones were generated that varied modestly in the level of WT-TDP43-eGFP-His, ΔNLS-TDP43-eGFP-His, or eGFP-His expression, and those with the most optimal eGFP level (high expression with normal cellular morphology and survival) were selected for further experiments.

### Subcellular Fractionation

Fractionation was used to examine the subcellular localization of TDP43 variant expression (Barmada et al., 2010; Archbold et al., 2018). Briefly, WT-TDP43-eGFP-His-, ΔNLS-TDP43-eGFP-His-, or eGFP-His-expressing HeLa cells were plated at a density of 1 × 10^5^ cells/well, incubated for 48 h, and then half of the plates were induced with1 μg/ml doxycycline for an additional 48 h. To efficiently capture cyTDP43-bound miRNAs, the stable cell lines were irradiated in a UVP HL-2000 HybriLinker UV cross-linker oven (Thermo Fisher Scientific) at 400 mJ/cm^2^ and again at 200 mJ/cm^2^ (King et al., 2014), and then cells were protected from light, detached by scraping in the buffer, and harvested by centrifugation (4 min, 1,000 rpm, 4°C). Cells were lysed as previously reported (Sharma et al., 2011; King et al., 2014) on ice for 5 min in resuspension buffer [RSB: 10 mM Tris (pH 7.4), 10 mM NaCl, 3 mM MgCl_2_, Complete Protease Inhibitor Cocktail (Roche), 0.2 U/μl RNase Inhibitor (cat# N8080119, Applied Biosystems, Thermo Fisher Scientific, Foster City, CA, USA), and DNase per the manufacturer’s instructions (cat# 1023460, Qiagen, Hilden, Germany)]. RSB supplemented with 0.6% IGEPAL (#CA-630, Sigma) was added to the lysate and incubated for 5 min before centrifugation (15 min, 2000 G, 4°C) to separate the cytosolic (supernatant) and nuclear (pellet) fractions. Imidazole (10 mM; #10250-25g, Sigma) was added to the cytoplasmic supernatant fraction. The nuclear pellet fraction was resuspended in RSB, centrifuged (10 min, 2000 G, 4°C), and then the resulting nuclear pellet was resuspended and sonicated in CHAPS lysis buffer (5 M NaCl, 1 M imidazole, 0.5 M Na_2_PO_4_, 1 M NaH_2_PO_4_, and 10% CHAPS, pH 8.0), incubated on ice for 30 min, and centrifuged (15 min, 6800 G, 4°C; Figueroa-Romero et al., 2009). An aliquot was removed at each step and total protein was measured using a Pierce BCA Protein Assay Kit (cat# 23227, Thermo Fisher Scientific) following the manufacturer’s instructions before mixing with 2× sample buffer [100 mM Tris (pH 6.8), 4% SDS, 10% glycerol, 0.015% bromophenol blue; Figueroa-Romero et al., 2009]. To validate the subcellular fractionation efficiency, samples were boiled for 5 min in 2× sample buffer and subjected to Western blot analysis.

### WT-TDP43-eGFP-His and ΔNLS-TDP43-eGFP-His Pull-Down

To further evaluate TDP43 variant expression patterns, WT-TDP43-eGFP-His, ΔNLS-TDP43-eGFP-His, and control eGFP-His were pulled down *via* their His tags from the subcellular fractions using nickel-agarose (Ni^2+^) beads (Qiagen; Figueroa-Romero et al., 2009). Briefly, 40 μl Ni^2+^-beads prewashed in CHAPS buffer were added to each 600 μl sample and rotated overnight at 4°C. Beads were then washed three times with CHAPS buffer and divided into two aliquots, one for protein and one for RNA extraction. A bead-only sample was included throughout the rest of the protocol as a negative control.

For protein extraction, bead aliquots were washed twice with CHAPS glycanase buffer (50 mM NaCl, 45 mM Na_2_HPO_4_, 5 mM NAH_2_PO_4_, 0.1% CHAPS, pH 8.0) and centrifuged between washes (15 s, max speed). Protein concentration was measured using a Pierce BCA Protein Assay Kit (cat# 23227, Thermo Fisher Scientific) following the manufacturer’s instructions, and the subcellular fractions were resuspended in 3× EDTA sample buffer (150 mM Tris (pH 6.8), 10 mM EDTA, 6% SDS, 15% glycerol, 0.0225% bromophenol blue) supplemented with 20 mM β-mercaptoethanol. Samples were incubated at 50°C for 20 min before Western blot analysis (Figueroa-Romero et al., 2009; King et al., 2014).

For extracting miRNAs, bead aliquots from the cytoplasmic fractions were washed twice with 1× PXL (0.1% SDS, 0.5% Triton X-100, 0.5% deoxycholate in 1× PBS) and twice with 5× PXL. Samples were then boiled for 3 min to reverse cross-linking (King et al., 2014), and QIAzol (Qiagen) was added and samples were stored at −80°C. Next, a miRNeasy Mini Kit (cat# 217004, Qiagen) was used to extract total RNA, enriched in cyTDP43-bound miRNAs. Purified RNA was treated with RNase-free DNase and stored at −80°C until required for cDNA synthesis or miRNA profiling.

### Western Blot

A 15 μg aliquot of protein per sample was resolved on a denaturing 10% polyacrylamide gel, and proteins were transferred to polyvinylidene difluoride (PVDF) membranes using a semidry blotter by established standard protocols (Figueroa-Romero et al., 2009; Lunn et al., 2009). Blots were probed overnight at 4°C with the following primary antibodies: rabbit anti-TDP43 (1:1,000; cat#A260, Cell Signaling Technology, Danvers, MA, USA), rabbit anti-histone 2B (H2B) member S (1:500; cat# NB100-56347, Novus Biologicals, Littleton, CO, USA), rabbit anti-GFP (1:1,000; cat#2555, Cell Signaling Technology), and rat anti-α-tubulin (1:5,000; cat#ab6160, Abcam). The next day, blots were rinsed and probed for 50 min at room temperature with appropriate HRP-conjugated secondary antibodies (1:1,000; Santa Cruz Biotechnology or New England Biolabs), then rinsed and visualized by enhanced chemiluminescence with Prime Western Blotting Detection Reagent (Amersham, GE Healthcare, Chicago, IL, USA).

### miRNA NanoString Profiling, Analysis, and Validation

To evaluate miRNA profiles associated with cyTDP43, total RNA from the cytoplasmic fractions pulled-down with Ni^2+^-beads for WT-TDP43-eGFP-His, ΔNLS-TDP43-eGFP-His, and eGFP-His lines (*n* = 4 for each line) was processed using the nCounter Human v3A miRNA Gene List (NanoString Technologies, Seattle, WA, USA) as published previously (Wohlfarth et al., 2017). A nCounter Digital Analyzer counted individual fluorescent barcodes that quantified and identified levels of up to 800 human-specific target miRNA molecules. nSolver Analysis Software 3.0 (NanoString Technologies) was used to background subtract and normalize the top 100 miRNA counts. Fold-change differences for WT-TDP43- or ΔNLS-TDP43-associated miRNAs relative to control (eGFP-His) were identified using *P* < 0.05 as the significance cutoff. Significant miRNAs were then analyzed using mirPath v.3^2^ (Vlachos et al., 2015) using a false discovery rate corrected *P*-value threshold of 0.05 and a microT threshold of 0.8 for predicted miRNA targets in the microT-CDS database to identify Kyoto Encyclopedia of Genes and Genomes (KEGG) pathways^3^ that associate with the cyTDP43-interacting miRNAs.

To validate the NanoString dataset, quantitative real-time PCR (qPCR) of select cyTDP43-associated miRNAs was performed using TaqMan Universal PCR Master Mix, TaqMan MicroRNA Assays (Thermo Fisher Scientific), and a 1:100 dilution of the pre-amplified NanoString analysis template as previously described (Figueroa-Romero et al., 2016) using the standard TaqMan protocol on a StepOnePlus Real-Time PCR System (Applied Biosystems). The cycle threshold (CT) values representing miRNA expression in the samples were calculated using StepOnePlus software and normalized to mir-92a-3p, a miRNA with a low coefficient of variation across samples. Next, ΔCT and ΔΔCT were calculated relative to the control group. miRNA levels were expressed as the mean ± standard error of the mean (SEM) for triplicates and statistically significant differences between either WT-TDP43-eGFP-His or ΔNLS-TDP43-eGFP-His to control (eGFP-His) were evaluated with a two-sample equal-variance Student’s *t*-test (Prism 5, GraphPad, San Diego, CA, USA); *P* < 0.05 was considered statistically significant.

## Results

### TDP43 Binds Differentially to Distinct miRNAs

**Figure 1:**
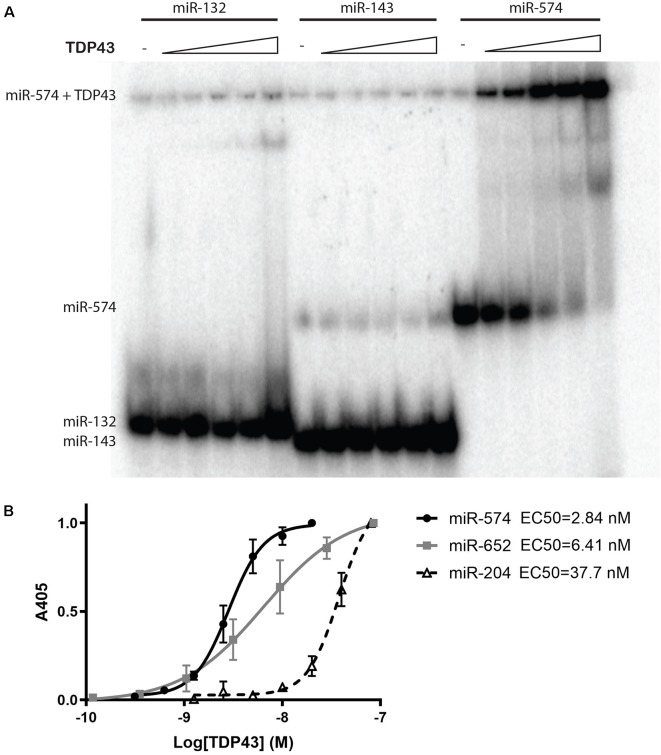
Trans-activation response element DNA/RNA-binding protein of 43 kDa (TDP43) binding specificity and apparent binding affinities for select microRNAs (miRNAs). **(A)** A native gel mobility shift assay was used to evaluate the association between TDP43 and previously associated miRNAs: miR-132, miR-143, and miR-574. Trace amounts of ^32^P-radiolabeled RNA (5 nM) were incubated with increasing concentrations of TDP43 (0, 0.05, 0.125, 0.25, 0.5, and 1.25 mM) and analyzed on a 12.5% native PAGE gel. A shift in band size was observed only for miR-574 (right). **(B)** Apparent binding affinities (EC50s) of TDP43 for select miRNAs with varying numbers of UG repeats were determined using a titration of biotinylated miRNAs in a colorimetric ELISA. EC50s are proportional to the number of UG repeats in each miRNA (miR-574, nine repeats > miR-652, three repeats > miR-204, two repeats).

To gain insight into TDP43′s ability to interact with specific miRNAs, we first used a native gel mobility shift assay to evaluate binding of denatured TDP43 to miR-132, miR-143, and miR-574, miRNAs all previously reported to associate with TDP43 (Buratti et al., 2010; Kawahara and Mieda-Sato, 2012; Freischmidt et al., 2013). Results demonstrated a band shift only for miR-574 (Figure 1A), with no observable shift for miR-132 or miR-143, suggesting that denatured TDP43 has a strong affinity only for miR-574. Since TDP43 binding often relies on associations with UG repeats (Ayala et al., 2005; Bhardwaj et al., 2013), the apparent binding affinity of TDP43 for miR-574 (9 UG repeats), miR-652 (3 UG repeats), and miR-204 (2 UG repeats) was next evaluated using a colorimetric ELISA to detect natively folded TDP43 bound to surface-captured biotinylated miRNAs (Rumora et al., 2010, 2013). EC50 values were determined for the binding of TDP43 to each miRNA from a titration of the biotinylated miRNAs. TDP43 had the highest apparent affinity for miR-574 (EC50 = 2.84 nM), a slightly lower binding affinity for miR-652 (EC50 = 6.41 nM), and the lowest binding affinity for miR-204 (EC50 = 37.7 nM; Figure 1B). These EC50s are proportional to the number of UG repeats in each miRNA sequence (miR-574 > miR-652 > miR-204) and support the contention that UG content dictates the relative binding affinity of TDP43 (Bhardwaj et al., 2013).

### Cells Expressing WT-TDP43-eGFP-His and ΔNLS-TDP43-eGFP-His Reflect Differential Subcellular TDP43 Localization

**Figure 2:**
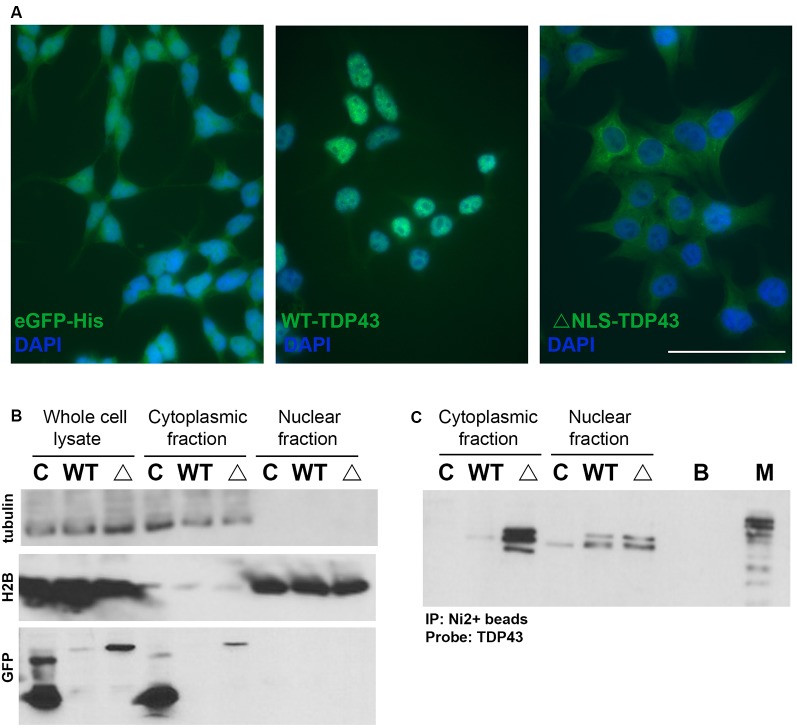
Doxycycline-inducible stable cell lines expressing TDP43 variants. **(A)** Representative images of control (eGFP-His), wild-type (WT)-TDP43-eGFP-His (WT-TDP43), and ΔNLS-TDP43-eGFP-His (ΔNLS-TDP43) stable HeLa cell lines depict primarily nuclear expression for WT-TDP43 and predominantly cytoplasmic expression for ΔNLS-TDP43. Scale bar = 50 μm. **(B)** Immunoblotting of whole-cell lysates and cytoplasmic and nuclear fractions for α-tubulin (cytoplasmic marker; top panel), H2B (nuclear marker; middle panel), and eGFP (a marker for expression of the eGFP-His-tagged doxycycline-inducible plasmids) following subcellular fractionation of the control (C), WT-TDP43-eGFP-His (WT), and ΔNLS-TDP43-eGFP-His (Δ) lines. eGFP immunoblotting demonstrates that eGFP-His (lower band) is expressed in the whole-cell lysates and cytoplasmic fraction of the control cell line, while eGFP-His-tagged TDP43 (upper band) is present in the whole-cell lysates and cytoplasmic fraction of the WT-TDP43-eGFP-His and ΔNLS-TDP43-eGFP-His cell lines, although only marginal expression seen in the WT-TDP43-eGFP-His cell line. **(C)** TDP43 immunoblotting following pull-down of the His-tagged proteins using Ni^2+^ beads. ΔNLS-TDP43-eGFP-His is detected mainly in the cytoplasmic fraction, with some expression also seen in the nuclear fraction, while WT-TDP43-eGFP-His is more highly abundant in the nuclear fraction, though modest expression is also seen in the cytoplasmic fraction. The slightly higher bands in both TDP43 lines represent the TDP43-eGFP-His fusion proteins, while the lower bands represent endogenous TDP43. In the cytoplasmic fraction, ΔNLS-TDP43-eGFP-His also exhibits a third band (lowest) that likely reflects a proteolytic cleavage product of TDP43. C = eGFP-His; WT = WT-TDP43-eGFP-His; Δ = ΔNLS-TDP43-eGFP-His; B = Ni^2+^ bead only control; M = marker/ladder.

To provide a model to study the effect of altered TDP43 cellular localization on miRNAs, we established doxycycline-inducible HeLa cell lines that stably express eGFP- and His-tagged WT-TDP43 or ΔNLS-TDP43, along with eGPF-His-expressing controls. Upon doxycycline-induced expression, eGFP imaging revealed primarily nuclear localization of overexpressed WT-TDP43-eGFP-His vs. primarily cytoplasmic localization of ΔNLS-TDP43-eGFP-His (Figure 2A). This differential localization was further supported by Western blotting of eGFP in nuclear and cytoplasmic fractions. α-Tubulin, a cytoplasmic protein, was detected in whole-cell lysates and cytoplasmic fractions for all three cell lines, as expected (Figure 2B, top panel). The nuclear marker H2B was present in whole-cell lysates and nuclear fractions of all three cell lines, and only weakly detected in their cytoplasmic fractions (Figure 2B, middle panel). eGFP expression was also confirmed in whole-cell lysates of all three cell lines (Figure 2B, bottom panel), although the expression levels were low in some fractions, so we enriched using a Ni^2+^-bead pull-down assay for His-tagged proteins to better evaluate subcellular localization in each fraction. The resulting data corroborated the primarily cytoplasmic localization of ΔNLS-TDP43-eGFP-His (Figure 2C) and verified the largely nuclear localization of WT-TDP43-eGFP-His that was accompanied by some anticipated cytoplasmic localization from its natural nucleus-cytoplasm shuttling role (Li et al., 2015; Weskamp and Barmada, 2018; Figure 2C). Of note, the lower of the two TDP43 bands in the nuclear fractions likely reflects endogenous TDP43 associating with the eGFP-His-tagged TDP43 aggregates, whereas the higher band represents the eGFP-His fusion protein. The third band in the ΔNLS-TDP43-eGFP-His cytoplasmic fraction most likely reflects a proteolytic TDP43 cleavage product (Li et al., 2015; Weskamp et al., 2020). Together, these data support our ability to efficiently separate the cytoplasmic and nuclear compartments for all three cell lines, with minimal inter-fraction contamination, and our ability to effectively pull down TDP43 *via* its His tag.

### WT-TDP43-eGFP-His- and ΔNLS-TDP43-eGFP-His-Expressing Cells Exhibit Differential miRNA Profiles

**Figure 3:**
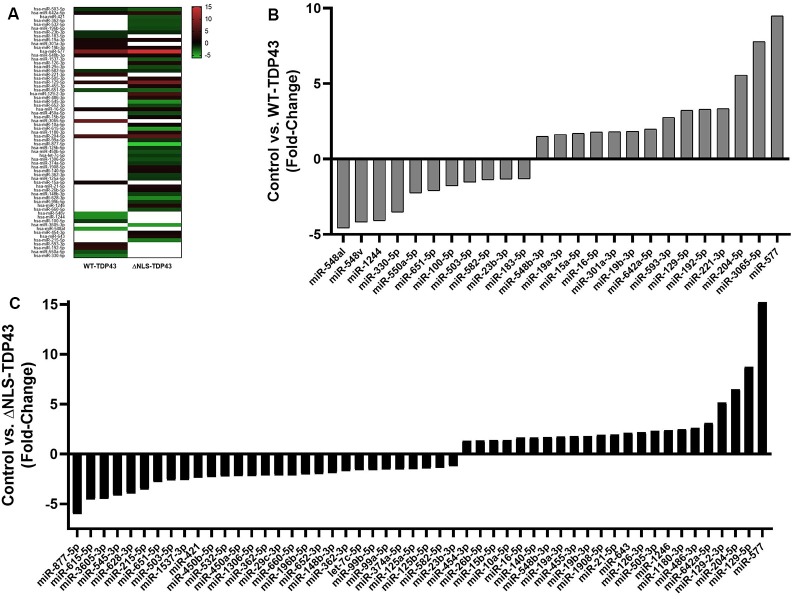
Enriched miRNAs associated with cyTDP43. miRNAs in the cytoplasmic fractions of the control (eGFP-His), WT-TDP43-eGFP-His (WT-TDP43), and ΔNLS-TDP43-eGFP-His (ΔNLS-TDP43) stable cell lines were profiled using NanoString technology. Each individual miRNA was normalized to the top 100 miRNAs and fold-change was determined relative to the control line; *n* = 4 per line. **(A)** Heat map of the fold-change of the top enriched miRNAs. Red represents positive values (increased enrichment), while green represents negative values (decreased enrichment) for the WT-TDP43 (left) and ΔNLS-TDP43 (right) lines relative to the control (eGFP-His) line. **(B,C)** Fold change of top enriched miRNAs in the cytoplasmic fraction of the WT-TDP43 **(B)** and ΔNLS-TDP43 **(C)**.

To identify miRNAs that interact with cytoplasmic WT-TDP43-eGFP-His or ΔNLS-TDP43-eGFP-His, we performed NanoString miRNA profiling of TDP43-bound miRNAs isolated by Ni^2+^-pulldown from cytoplasmic fractions of the respective stable HeLa cell lines. Appreciable target detection occurred for most miRNAs across all samples, and assessment of fold-changes vs. the control eGFP-His line identified 65 significantly altered cyTDP43-associated miRNAs (Figure 3 and Table 1). For WT-TDP43-eGFP-His cells, 11 miRNAs decreased and 14 increased in level (Figure 3B). In the ΔNLS-TDP43-eGFP-His cell line with primarily cytoplasmic TDP43 localization, more miRNAs satisfied the criteria for significant fold-enrichment, with 29 decreased and 23 increased in levels (Figure 3C). Interestingly, nine miRNAs (four decreased and five increased) in the WT-TDP43-eGFP-His cells and 11 miRNAs (six decreased and five increased) in the ΔNLS-TDP43-eGFP-His cells were enriched by more than 3-fold. Moreover, 12 miRNAs were present in both cell lines and exhibited similar over- or under-enrichment.

**Table 1:** Top enriched miRNAs.

	ΔNLS-TDP43	WT-TDP43
hsa-let-7c-5p	−1.63
hsa-miR-100-5p		−1.78
hsa-miR-10a-5p	1.40
hsa-miR-1180-3p	2.51
**hsa-miR-1244**		**−4.10**
hsa-miR-1246	2.39
hsa-miR-125a-5p	−1.52
hsa-miR-125b-5p	−1.45
hsa-miR-126-3p	2.18
**hsa-miR-129-2-3p**	**5.17**
**hsa-miR-129-5p**	**8.74**	**3.23**
hsa-miR-1306-5p	−2.21	
hsa-miR-140-5p	1.66	
hsa-miR-148b-3p	−1.90	
hsa-miR-1537-3p	−2.62	
hsa-miR-15a-5p		1.69
hsa-miR-15b-5p	1.39
hsa-miR-16-5p	1.64	1.78
hsa-miR-183-5p		−1.32
hsa-miR-1908-5p	1.91
hsa-miR-192-5p		3.30
hsa-miR-196b-5p	−2.05
hsa-miR-19a-3p	1.77	1.62
hsa-miR-19b-3p	1.80	1.83
**hsa-miR-204-5p**	**6.50**	**5.56**
**hsa-miR-215-5p**	**−3.57**
hsa-miR-21-5p	1.95
**hsa-miR-221-3p**		**3.34**
hsa-miR-23b-3p	−1.23	−1.35
hsa-miR-26b-5p	1.36
hsa-miR-29c-3p	−2.15
hsa-miR-301a-3p		1.80
**hsa-miR-3065-5p**		**7.79**
**hsa-miR-330-5p**		**−3.52**
hsa-miR-3605-3p	−4.51
hsa-miR-362-3p	−1.70
hsa-miR-362-5p	−2.16
hsa-miR-374a-5p	−1.54	
hsa-miR-421	−2.36
hsa-miR-450a-5p	−2.23
hsa-miR-450b-5p	−2.30
hsa-miR-454-3p	1.34
hsa-miR-455-3p	1.79
hsa-miR-486-3p	2.61
hsa-miR-503-5p	−2.64	−1.54
hsa-miR-505-3p	2.32
hsa-miR-532-5p	−2.25
**hsa-miR-545-3p**	**−4.16**
**hsa-miR-548al**		**−4.58**
hsa-miR-548b-3p	1.71	1.50
**hsa-miR-548v**		**−4.19**
hsa-miR-550a-5p		−2.26
**hsa-miR-577**	**15.23**	**9.49**
hsa-miR-582-5p	−1.37	−1.38
hsa-miR-593-3p		2.76
**hsa-miR-615-5p**	**−4.57**
**hsa-miR-628-3p**	**−3.95**
hsa-miR-642a-5p*	**3.11**	1.99
hsa-miR-643	2.13
hsa-miR-651-5p	−2.81	−2.10
hsa-miR-652-3p	−2.00
hsa-miR-660-5p	−2.14
**hsa-miR-877-5p**	**−6.03**
hsa-miR-99a-5p	−1.55
hsa-miR-99b-5p	−1.60

*Bold denotes fold changes ≥3; shading indicates miRNAs altered in both cell lines*.

**Figure 4:**
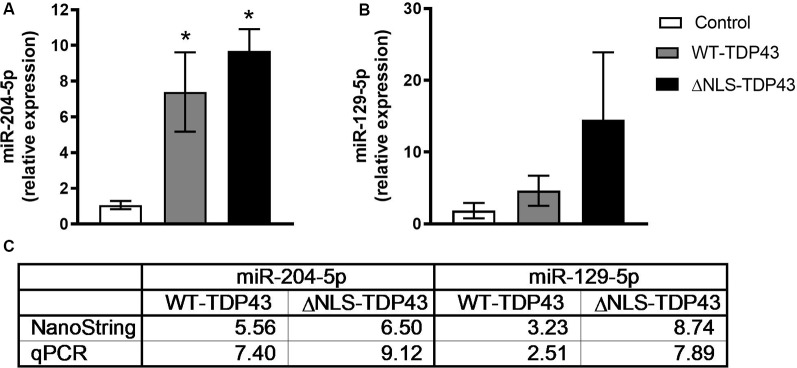
Confirmation of select enriched miRNAs. Levels of select miRNAs from the NanoString profiling results in cytoplasmic fractions of the control (eGFP-His), WT-TDP43-eGFP-His (WT-TDP43), and ΔNLS-TDP43-eGFP-His (ΔNLS-TDP43) lines were confirmed using quantitative real-time PCR (qPCR). Results were normalized to miR-92a (ΔCT) and then relative to the control group (ΔΔCT) and expressed as mean fold-change ± standard error of the mean (SEM; *n* = 4 per line). **(A)** miR-204-5p was significantly upregulated relative to control in both TDP43 lines in a similar proportion to the NanoString results (*p < 0.05). **(B)** miR-129-5p levels in both TDP43 lines were upregulated at fold-changes equivalent to the NanoString results, but did not reach significance relative to the control line. **(C)** Fold-change comparisons between the qPCR and NanoString profiling results.

To validate the NanoString profiling results, we performed qPCR on the same samples used for miRNA profiling for miR-204-5p and miR-129-5p. qPCR miRNA levels were normalized to miR-92a, which has a low coefficient of variation relative to other miRNAs in all cell lines (data not shown). Similar to the NanoString data, qPCR showed statistically significant increases in miR-204-5p and levels in both WT-TDP43-eGFP-His and ΔNLS-TDP43-eGFP-His HeLa cell lines (Figures 4A,C). Likewise, miR-129-5p increased in both WT-TDP43-eGFP-His and ΔNLS-TDP43-eGFP-His cell lines but did not reach statistical significance (Figures 4B,C).

**Figure 5:**
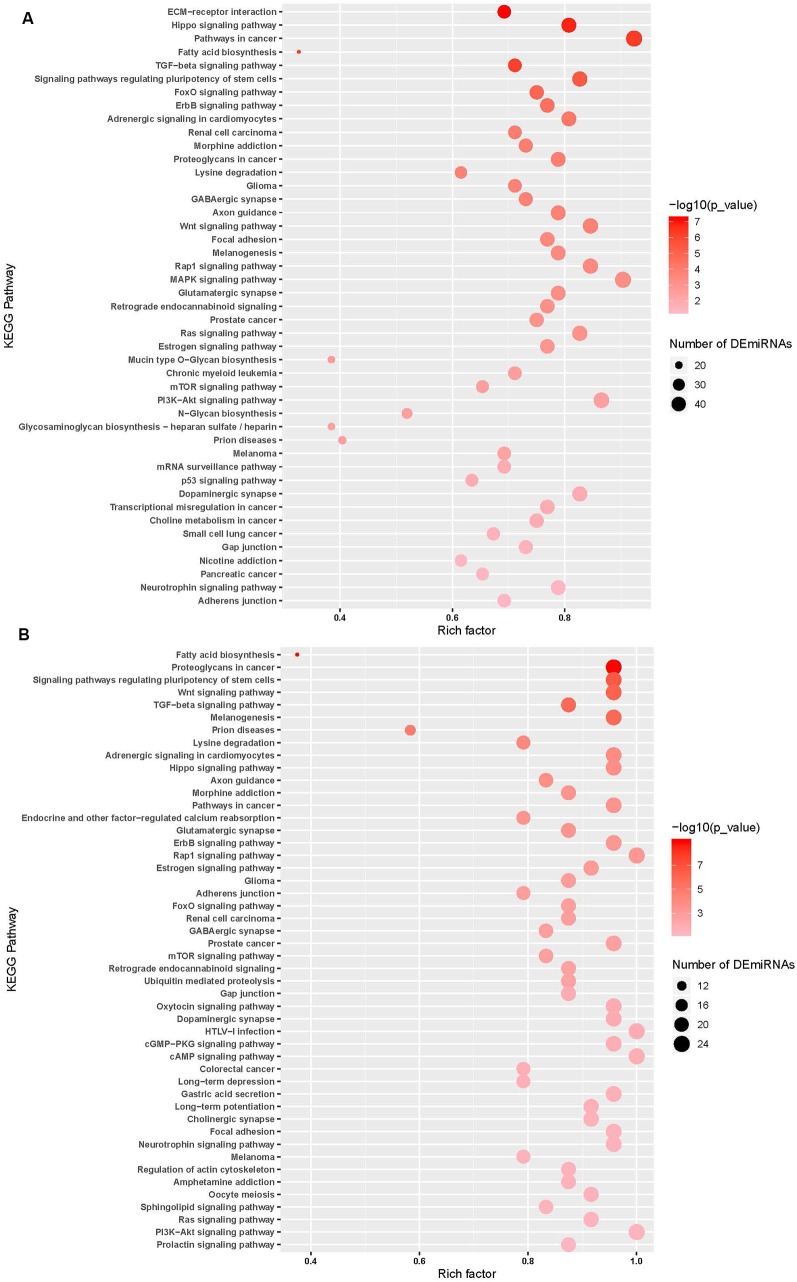
miRNA pathway analysis of the top enriched miRNAs. Predicted gene targets and associated KEGG pathways following pathway analysis of the top ΔNLS-TDP43 **(A)** and WT-TDP43 **(B)** miRNAs using mirPath v.3. Higher intensity of red shading reflects increasing significance values, while the size of the nodes represents the number of altered miRNAs within the pathway. Lists of the predicted pathways represented among the ΔNLS-TDP43 and WT-TDP43 enriched miRNAs are also detailed in Supplementary Tables S1, S2.

miRNA pathway analysis of the 65 miRNAs using mirPath v.3 (Vlachos et al., 2015) further revealed several predicted gene targets and associated KEGG pathways that offered insight into the relevance of the identified cyTDP43-interacting miRNAs. Pathways represented in both WT-TDP43-eGFP-His and ΔNLS-TDP43-eGFP-His included multiple cancer and neuronal function and health pathways, such as GABAergic and glutamatergic synapses, axon guidance, and neurotrophin signaling pathways (Figure 5; Supplementary Tables S1, S2). Taken together, these data indicate that TDP43 expression and localization significantly affects the levels of multiple miRNAs, which are predicted to affect several genes and biological pathways that are relevant to nervous system health and disease.

## Discussion

TDP43 mutations are linked to ALS (Kapeli et al., 2017), and cyTDP43 is a universal histopathological hallmark of *postmortem* brain and spinal cord tissue from fALS and sALS subjects (Neumann et al., 2006; Zhang et al., 2008; Liu-Yesucevitz et al., 2010). However, the implications of these cytoplasmic inclusions on key cellular processes, including gene expression, have not been fully elucidated. TDP43 is central to miRNA biogenesis, and its ability to directly interact with miRNAs regulates expression of numerous genes (Kawahara and Mieda-Sato, 2012; King et al., 2014; Emde et al., 2015; Loffreda et al., 2015; Paez-Colasante et al., 2015). We hypothesized that one consequence of TDP43 cytoplasmic translocation is aberrant protein/miRNA interactions that may ultimately affect cell function and viability through epigenetic mechanisms. To address this idea, we first showed that TDP43 exhibits differential binding affinity for specific miRNAs and that binding affinities correlate with the number of miRNA UG repeats. This suggests that cyTDP43 may preferentially affect certain miRNAs during disease pathogenesis. Next, using stable cell lines expressing inducible TDP43 variants with primarily nuclear (WT-TDP43-eGFP-His) or cytoplasmic (ΔNLS-TDP43-eGFP-His) subcellular localization, we identified 65 differentially enriched miRNAs that associate with cyTDP43, including known and novel hits that potentially regulate multiple predicted genes and pathways. Together, these findings provide insight into the consequences of TDP43 mislocalization in ALS by identifying specific miRNAs that associate with cyTDP43. These data could ultimately shed light on pathogenic mechanisms, biomarkers, and reversible ALS treatment targets.

TDP43 is normally predominantly found in the nucleus; however, ALS-associated mutations and/or cellular stress can prompt hyper-phosphorylation and sequestration into insoluble, ubiquitin-positive, denatured cytoplasmic aggregates (Neumann et al., 2006, 2009). Previous studies also indicate that TDP43 knockdown is toxic (Sephton et al., 2010; Iguchi et al., 2013; Barmada et al., 2015), that TDP43 toxicity more closely correlates with increased cyTDP43 than with the level of nuclear TDP43 (Barmada et al., 2010), and that cyTDP43 clearance can exert beneficial effects on neuronal survival (Barmada et al., 2014) and functional outcomes (Walker et al., 2015). cyTDP43 has thus been the focus of multiple *in vitro* and *in vivo* studies in recent years (Miguel et al., 2011; Walker et al., 2015; Birsa et al., 2020). Of particular relevance is the rNLS8 ALS mouse that exhibits inducible expression of ΔNLS-TDP43 in motor neurons. This transgenic mouse recapitulates many of the pathologic characteristics of ALS, including cyTDP43 aggregation, motor neuron death, increases in CSF neurofilament levels, neuromuscular junction loss, muscle atrophy, and abnormal compound muscle axon potentials measured by electromyogram (Spiller et al., 2016a,b, 2018, 2019). However, a complete understanding of the effect TDP43 mislocalization has on specific miRNAs is lacking. This is important for neuronal health because cytoplasmic TDP43 aggregates can interact with and sequester key miRNAs, thus limiting their ability to perform their normal regulatory functions. Cytoplasmic TDP43 aggregates also impair normal miRNA maturation through a loss of nuclear localization and interactions with miRNA biosynthetic pathway components (Barmada and Finkbeiner, 2010; Kawahara and Mieda-Sato, 2012; Weskamp and Barmada, 2018). Herein, we specifically focused on understanding how cytoplasmic translocation of TDP43 affects miRNA dynamics as another potential pathomechanism.

Before examining the impact of cyTDP43 on miRNAs, we first assessed TDP43 interactions and binding affinities to three specific miRNAs known to associate with TDP43: miR-132, miR-143, and miR-574-5p (Buratti et al., 2010; Kawahara and Mieda-Sato, 2012; Freischmidt et al., 2013). We found that denatured TDP43 complexed with miR-574-5p, a miRNA first predicted to bind to TDP43 based on its UG-repeat regions in primary sequence (Buratti et al., 2010), but not miR-132 or miR-143, within the detection limit of our native gel mobility shift assay. We further verified that apparent binding affinities of TDP43 to miRNAs increased relative to the number of UG repeats; miR-574-5p with nine UG repeats had the highest affinity relative to miRNAs with only two or three UG repeats. Though all three miRNAs we selected to examine are known to exhibit altered levels in CSF and serum from ALS subjects (Freischmidt et al., 2013), we did not observe interactions between TDP43 and miR-132 or miR-143. This may be related to the use of denatured TDP43 in our assay, selected because of its biological representation of TDP43 aggregates, or due to differential regulation of the miRNAs in the CSF and serum vs. cells, suggesting that cell-type-specific mechanisms may be involved (Freischmidt et al., 2013). Alternatively, TDP43 can bind UG-rich sequences as well as other secondary structures in pri- and pre-miRNAs (Kawahara and Mieda-Sato, 2012). Nonetheless, our data support the idea that TDP43 localization can differentially affect specific miRNAs.

To better understand the role of TDP43 mislocalization on miRNA dynamics, we created stable inducible cell lines that express predominantly nuclear (WT) or cytoplasmic (ΔNLS) TDP43 variants in HeLa cells, which are a known and reliable model of TDP43 pathology (Ayala et al., 2008; Ling et al., 2010; Gu et al., 2017). cyTDP43 expression was regulated by a doxycycline-inducible promoter to prevent cytotoxicity from the overexpressed or mutant proteins (Walker et al., 2015), a GFP tag facilitated visualization, and a His-tag was present to leverage cyTDP43/miRNA profiling using a pulldown assay. We confirmed moderate WT-TDP43 overexpression that led to primarily nuclear expression and marginal cytoplasmic expression, similar to other WT-TDP43 overexpression models (Neumann et al., 2006, 2009; Barmada et al., 2015; Li et al., 2015; Wang et al., 2015). We similarly verified that the mutant ΔNLS-TDP43 was localized to the cytoplasm, leading to diffuse or aggregated cyTDP43 as in previous *in vitro* cell models (Winton et al., 2008; Barmada et al., 2010; Liu-Yesucevitz et al., 2010), *in vivo* animal models (Wils et al., 2010; Miguel et al., 2011; Walker et al., 2015), and human *postmortem* neuronal tissue (Neumann et al., 2006; Zhang et al., 2008; Liu-Yesucevitz et al., 2010; Davidson et al., 2016).

Importantly, since cyTDP43 pathology is present in both fALS and sALS (Hardiman et al., 2017), and even a minor increase in TDP43 levels can drive neurodegeneration (Barmada and Finkbeiner, 2010; Barmada et al., 2010; Wegorzewska and Baloh, 2011; Janssens et al., 2013; Weskamp and Barmada, 2018), miRNAs associated with cyTDP43 from either cell line could potentially provide insight into mechanisms underlying miRNA dynamics in ALS. miRNAs that interacted with cyTDP43 were assessed by NanoString profiling, an approach that enables rapid, accurate analysis of up to 800+ biologically relevant miRNAs without the need for amplification (M’Boutchou and van Kempen, 2016,^4^). We identified 65 miRNAs that significantly differentially associated with cyTDP43 vs. control eGFP-His-expressing cells. As expected, cyTD43 expression in the ΔNLS-TDP43-eGFP-His line led to a larger number of associated miRNAs (*n* = 52) than in the WT-TDP43-eGFP-His line (*n* = 25), which were increased and decreased in enrichment relative to the control cell line. Interestingly, 11 miRNAs varied by 3-fold or more, and 12 miRNAs were altered in both the WT-TDP43-eGFP-His and ΔNLS-TDP43-eGFP-His lines.

Among the most highly differentially represented miRNAs, we identified several novel miRNAs that have not previously been linked to ALS, such as miR-3065 and miR-129-5p. While little has been reported on the function of miR-3065, miR-129-5p is implicated in epilepsy (Sosanya et al., 2015; Liu et al., 2017; Rajman et al., 2017), neuroinflammation in ischemia-reperfusion (Li et al., 2017), and Alzheimer’s disease (Zeng et al., 2019). Our study suggests a potential neuroprotective role for miR-129-5p, which is blocked when miR-129-5p associates with cyTDP43; however, more research is needed to fully understand the meaning of the interaction of miR-129-5p with cyTDP43.

Several of the cyTDP43-associated miRNAs we identified herein are known to have a role in ALS pathogenesis. miR-577, which was highly increased in both cells lines in our study, is predicted to target eukaryotic translation initiation factor 2C, 2/argonaute 2 (EIF2C2/AGO2), a member of the RNA-induced silencing complex (RISC), which interacts with mature miRNAs to bind mRNAs and interfere with their translation (Ha and Kim, 2014; Kobayashi and Tomari, 2016). Interestingly, miR-577 is decreased in *postmortem* sALS spinal cord tissue with a concomitant increase in EIF2C2/AGO2 gene expression (Figueroa-Romero et al., 2016). In parallel, we found that miR-129-2-3p was highly associated with cyTDP43 in our ΔNLS-TDP43-eGFP-His line and that miR-204-5p was increased in both lines. However, both miR-129-2-3p and miR-204-5p are lower at the neuromuscular junction of ALS subjects (De Felice et al., 2018). Finally, miR-221-3p was among the more highly enriched cy-TDP43-associated miRNAs in our study but has been reported to be both increased and decreased in other ALS studies (D’Erchia et al., 2017; Di Pietro et al., 2018; Taguchi and Wang, 2018).

The discordance between reported studies and some of our top miRNAs likely reflects that the clinical profiling studies evaluated case/control differences in plasma, blood, CSF, muscle biopsies, or spinal cord tissue (Joilin et al., 2019), while we looked more directly at miRNAs that associate with cyTDP43. It is possible that miRNAs that more highly associate with cyTDP43 may be sequestered and do not reach peripheral biofluids or tissues, and thereby are reduced in studies profiling biofluids and tissue samples. It is also plausible that miRNAs that interact with cyTDP43 earlier in the course of the disease could lead to ultimate depletion and/or compensatory changes in *postmortem* assessment.

Another confounding factor is that TDP43 autoregulates its expression levels (Ayala et al., 2011; Prasad et al., 2019). TDP43 is known to localize to stress granules under stress conditions (Emde et al., 2015; Paez-Colasante et al., 2015; Weskamp and Barmada, 2018; Chen and Cohen, 2019), and cytoplasmic TDP43 redistribution may represent a physiologic response to stress that becomes toxic if the normal distribution is not reestablished (Barmada and Finkbeiner, 2010). Thus, it is unclear if the association of certain miRNAs with cyTDP43 represents a possible preventive measure or rather promotes neuronal injury. In the case of miR-204, levels are increased in a cellular model of Parkinson’s disease (Talepoor Ardakani et al., 2019), it is implicated in optic nerve injury (Wang et al., 2018), and miR-204 overexpression impairs neurite outgrowth (López-González et al., 2018) while inhibition provides neuroprotection (Yan et al., 2019). Ultimately, more research is needed to determine how miRNA profiles change over time, associate with TPD43, and impact ALS progression.

We also found an increased association of miR-15a-5p and miR-16-5p with cyTDP43. These miRNAs belong to a miR-15/16 cluster in an intronic region of the DLEU2 gene on chromosome 13 and are linked to ALS through profiling peripheral blood, neuron-derived extracellular vesicles, and muscle from ALS subjects (Liguori et al., 2018; Si et al., 2018; Katsu et al., 2019). Much of the published data on this cluster indicate that miR-15/16 members directly target the apoptosis regulator Bcl-2 to inhibit cellular proliferation, induce cancer cell apoptosis, and thereby reduce tumorigenicity (Aqeilan et al., 2010; Pekarsky et al., 2018). Since Bcl-2 is also tied to apoptotic regulation in neural cells (Azzouz et al., 2000; Soane and Fiskum, 2005; Hollville et al., 2019), similar mechanisms could be relevant to ALS. We speculate that the increased associations of miR-15/16 we observed with cyTDP43 could sequester these miRNAs, preventing Bcl-2 regulation, which in turn could promote cell death in ALS. Future studies are needed to confirm this possibility and understand the role of miR-15/16 in ALS pathogenesis.

At the pathway level, our results overlap with reported studies. Examining predicted target pathways identified over 30 linked to both WT-TDP43-eGFP-His and ΔNLS-TDP43-eGFP-His lines, as well as several others that were represented in either cell line. While many were related to cancer signaling, pathways connected to neuronal function and health were also commonly reflected, including GABAergic and glutamatergic synapses, axon guidance, and neurotrophin signaling pathways. Moreover, our analysis identified several pathways linked to miRNA profiles identified in ALS subject muscle tissue and serum, including regulation of actin cytoskeleton, ubiquitin-mediated proteolysis, and transforming growth factor-β (TGF-β) signaling (Kovanda et al., 2018; Taguchi and Wang, 2018). The TGF-β signaling pathway is an established player in neuronal health (Barmada and Finkbeiner, 2010; Katsuno et al., 2011) and increased levels are associated with early disease stages in animal models and ALS subjects (Si et al., 2014, 2015; Meroni et al., 2019). Thus, these and other related pathways may provide important insights into the mechanisms and therapeutic opportunities for ALS.

Although the current study identified several intriguing cyTDP43-associated miRNAs and predicted pathways, we acknowledge that these data are based on an *in vitro* system with mutant cyTDP43 expression. The ΔNLS-TDP43 mutation, however, offers an important well-established strategy to model ALS and examine both disease mechanisms and resultant phenotypic consequences of cyTDP43 expression (Winton et al., 2008; Barmada et al., 2010; Miguel et al., 2011; Walker et al., 2015). Notably, *in vitro* studies using similar strategies have also verified that toxicity associated with ΔNLS-TDP43 parallels that of common TDP43 mutations seen in ALS subjects (Winton et al., 2008; Barmada et al., 2010, 2014). The overlap between our ΔNLS-TDP43 results and those from the WT-TDP43 line exhibiting moderate overexpression further supports the relevance of our observations. Future studies examining miRNA profiles that associate with cyTDP43 in relevant ALS animal models (Walker et al., 2015) and *postmortem* tissue are needed to both confirm the *in vitro* data as well as assess potential interventions based on identified targets.

We also acknowledge that cyTDP43 translocation can have implications beyond direct interactions with mature miRNAs. Our initial assessments of pri-miRNA levels for two differentially expressed mature miRNAs observed in ALS subject *postmortem* spinal cord tissue indicates that pri-miRNA expression was not altered, but that alterations must emerge at the level of miRNA processing (Figueroa-Romero et al., 2016). Other studies have also verified that TDP43 directly interacts with pri-miRNA and pre-miRNA intermediates and miRNA biosynthetic pathway components (Buratti et al., 2010; Kawahara and Mieda-Sato, 2012). Thus, while in the current study we only assessed mature miRNAs that interacted with TDP43, it is possible the impact of TDP43 may be conferred on miRNA intermediates along the biogenesis pathway or *via* sequestration in inclusions like stress granules (Emde et al., 2015; Paez-Colasante et al., 2015; Weskamp and Barmada, 2018). Future assessment of these alternative roles of cyTDP43 is needed. Finally, the miRNAs we identified did not overlap entirely with other reported studies (Dardiotis et al., 2018). This may be due to the heterogeneity in sALS samples examined by other studies relative to our specific focus on miRNAs bound to cyTDP43 in HeLa cells.

Overall, the current study used cellular models to identify miRNAs that interact with cyTDP43. We identified known and novel dysregulated miRNAs, data that provide a foundation for inferred biological insight and potential pathomechanisms from predicted pathways. cyTDP43 inclusions, by sequestering miRNAs, may skew neuronal epigenetic homeostasis and result in motor neuron death. Our findings may therefore guide further research examining the molecular downstream events resulting from dysregulated miRNAs that influence neurodegeneration in ALS and identify new biomarkers and therapeutic targets for disease diagnosis and intervention.

## Data Availability Statement

The datasets generated for this study can be found in the Gene Expression Omnibus (GEO; https://www.ncbi.nlm.nih.gov/geo/query/acc.cgi?acc=GSE145214).

## Author Contributions

XP-C, CF-R, JH, LH, NW, SB, and EF contributed to the conception and design of the study. XP-C, CF-R, AR, FM, JMH, CB, GT, and LH contributed to the acquisition of data. XP-C and JH performed the statistical analysis. XP-C, C-FR, AR, JH, LH, SB, SS, and EF interpreted the data. XP-C and SS wrote the first draft of the manuscript. CF-R and AR wrote sections of the manuscript. All authors contributed to manuscript revision, read, and approved the submitted version.

## Conflict of Interest

s EF consulted for Novartis in 2019. The remaining authors declare that the research was conducted in the absence of any commercial or financial relationships that could be construed as a potential conflict of interest.

## Footnotes

^1^https://brcf.medicine.umich.edu/cores/advanced-genomics/

^2^http://snf-515788.vm.okeanos.grnet.gr/

^3^http://www.genome.jp/kegg/

^4^https://www.nanostring.com/products/mirna-assays

## References

[B1] Aqeilan R. I. Calin G. A. Croce C. M. (2010). miR-15a and miR-16–1 in cancer: discovery, function and future perspectives. Cell Death Differ. 17, 215–220. 10.1038/cdd.2009.69 19498445

[B2] Archbold H. C. Jackson K. L. Arora A. Weskamp K. Tank E. M. Li X.. (2018). TDP43 nuclear export and neurodegeneration in models of amyotrophic lateral sclerosis and frontotemporal dementia. Sci. Rep. 8:4606. 10.1038/s41598-018-22858-w 29545601 PMC5854632

[B3] Ayala Y. M. De Conti L. Avendaño-Vázquez S. E. Dhir A. Romano M. D’Ambrogio A.. (2011). TDP-43 regulates its mRNA levels through a negative feedback loop. EMBO J. 30, 277–288. 10.1038/emboj.2010.310 21131904 PMC3025456

[B4] Ayala Y. M. Pantano S. D’Ambrogio A. Buratti E. Brindisi A. Marchetti C.. (2005). Human, *Drosophila*, and C.elegans TDP43: nucleic acid binding properties and splicing regulatory function. J. Mol. Biol. 348, 575–588. 10.1016/j.jmb.2005.02.038 15826655

[B5] Ayala Y. M. Zago P. D’Ambrogio A. Xu Y. F. Petrucelli L. Buratti E.. (2008). Structural determinants of the cellular localization and shuttling of TDP-43. J. Cell Sci. 121, 3778–3785. 10.1242/jcs.038950 18957508

[B6] Azzouz M. Hottinger A. Paterna J. C. Zurn A. D. Aebischer P. Büeler H. (2000). Increased motoneuron survival and improved neuromuscular function in transgenic ALS mice after intraspinal injection of an adeno-associated virus encoding Bcl-2. Hum. Mol. Genet. 9, 803–811. 10.1093/hmg/9.5.803 10749988

[B7] Balendra R. Isaacs A. M. (2018). C9orf72-mediated ALS and FTD: multiple pathways to disease. Nat. Rev. Neurol. 14, 544–558. 10.1038/s41582-018-0047-2 30120348 PMC6417666

[B8] Barmada S. J. Finkbeiner S. (2010). Pathogenic TARDBP mutations in amyotrophic lateral sclerosis and frontotemporal dementia: disease-associated pathways. Rev. Neurosci. 21, 251–272. 10.1515/revneuro.2010.21.4.251 21086759

[B9] Barmada S. J. Ju S. Arjun A. Batarse A. Archbold H. C. Peisach D.. (2015). Amelioration of toxicity in neuronal models of amyotrophic lateral sclerosis by hUPF1. Proc. Natl. Acad. Sci. U S A 112, 7821–7826. 10.1073/pnas.1509744112 26056265 PMC4485101

[B10] Barmada S. J. Serio A. Arjun A. Bilican B. Daub A. Ando D. M.. (2014). Autophagy induction enhances TDP43 turnover and survival in neuronal ALS models. Nat. Chem. Biol. 10, 677–685. 10.1038/nchembio.1563 24974230 PMC4106236

[B11] Barmada S. J. Skibinski G. Korb E. Rao E. J. Wu J. Y. Finkbeiner S. (2010). Cytoplasmic mislocalization of TDP-43 is toxic to neurons and enhanced by a mutation associated with familial amyotrophic lateral sclerosis. J. Neurosci. 30, 639–649. 10.1523/JNEUROSCI.4988-09.2010 20071528 PMC2821110

[B12] Bhardwaj A. Myers M. P. Buratti E. Baralle F. E. (2013). Characterizing TDP-43 interaction with its RNA targets. Nucleic Acids Res. 41, 5062–5074. 10.1093/nar/gkt189 23519609 PMC3643599

[B13] Birsa N. Bentham M. P. Fratta P. (2020). Cytoplasmic functions of TDP-43 and FUS and their role in ALS. Semin. Cell Dev. Biol. 99, 193–201. 10.1016/j.semcdb.2019.05.023 31132467

[B14] Brown R. H. Al-Chalabi A. (2017). Amyotrophic lateral sclerosis. N. Engl. J. Med. 377, 162–172. 10.1056/NEJMra1603471 28700839

[B15] Buratti E. De Conti L. Stuani C. Romano M. Baralle M. Baralle F. (2010). Nuclear factor TDP-43 can affect selected microRNA levels. FEBS J. 277, 2268–2281. 10.1111/j.1742-4658.2010.07643.x 20423455

[B16] Campos-Melo D. Droppelmann C. A. He Z. Volkening K. Strong M. J. (2013). Altered microRNA expression profile in Amyotrophic Lateral Sclerosis: a role in the regulation of NFL mRNA levels. Mol. Brain 6:26. 10.1186/1756-6606-6-26 23705811 PMC3668997

[B17] Chen Y. Cohen T. J. (2019). Aggregation of the nucleic acid-binding protein TDP-43 occurs *via* distinct routes that are coordinated with stress granule formation. J. Biol. Chem. 294, 3696–3706. 10.1074/jbc.RA118.006351 30630951 PMC6416430

[B18] Chen-Plotkin A. S. Lee V. M. Trojanowski J. Q. (2010). TAR DNA-binding protein 43 in neurodegenerative disease. Nat. Rev. Neurol. 6, 211–220. 10.1038/nrneurol.2010.18 20234357 PMC2892118

[B19] Chia R. Chiò A. Traynor B. J. (2018). Novel genes associated with amyotrophic lateral sclerosis: diagnostic and clinical implications. Lancet Neurol. 17, 94–102. 10.1016/s1474-4422(17)30401-5 29154141 PMC5901717

[B20] Dardiotis E. Aloizou A. M. Siokas V. Patrinos G. P. Deretzi G. Mitsias P.. (2018). The role of MicroRNAs in patients with amyotrophic lateral sclerosis. J. Mol. Neurosci. 66, 617–628. 10.1007/s12031-018-1204-1 30415446

[B21] Davidson Y. Robinson A. C. Liu X. Wu D. Troakes C. Rollinson S.. (2016). Neurodegeneration in frontotemporal lobar degeneration and motor neurone disease associated with expansions in C9orf72 is linked to TDP-43 pathology and not associated with aggregated forms of dipeptide repeat proteins. Neuropathol. Appl. Neurobiol. 42, 242–254. 10.1111/nan.122921 26538301 PMC4832296

[B22] De Felice B. Manfellotto F. Fiorentino G. Annunziata A. Biffali E. Pannone R.. (2018). Wide-ranging analysis of MicroRNA profiles in sporadic amyotrophic lateral sclerosis using next-generation sequencing. Front. Genet. 9:310. 10.3389/fgene.2018.00310 30154826 PMC6102490

[B23] D’Erchia A. M. Gallo A. Manzari C. Raho S. Horner D. S. Chiara M.. (2017). Massive transcriptome sequencing of human spinal cord tissues provides new insights into motor neuron degeneration in ALS. Sci. Rep. 7:10046. 10.1038/s41598-017-10488-7 28855684 PMC5577269

[B24] Di Carlo V. Grossi E. Laneve P. Morlando M. Dini Modigliani S. Ballarino M.. (2013). TDP-43 regulates the microprocessor complex activity during *in vitro* neuronal differentiation. Mol. Neurobiol. 48, 952–963. 10.1007/s12035-013-8564-x 24113842

[B25] Di Pietro L. Lattanzi W. Bernardini C. (2018). Skeletal muscle MicroRNAs as key players in the pathogenesis of amyotrophic lateral sclerosis. Int. J. Mol. Sci. 19:E1534. 10.3390/ijms19051534 29786645 PMC5983603

[B26] Dolati S. Marofi F. Babaloo Z. Aghebati-Maleki L. Roshangar L. Ahmadi M.. (2018). Dysregulated network of miRNAs involved in the pathogenesis of multiple sclerosis. Biomed. Pharmacother. 104, 280–290. 10.1016/j.biopha.2018.05.050 29775896

[B27] Emde A. Eitan C. Liou L. L. Libby R. T. Rivkin N. Magen I.. (2015). Dysregulated miRNA biogenesis downstream of cellular stress and ALS-causing mutations: a new mechanism for ALS. EMBO J. 34, 2633–2651. 10.15252/embj.201490493 26330466 PMC4641530

[B28] Engels B. M. Hutvagner G. (2006). Principles and effects of microRNA-mediated post-transcriptional gene regulation. Oncogene 25, 6163–6169. 10.1038/sj.onc.1209909 17028595

[B29] Figueroa-Romero C. Hur J. Lunn J. S. Paez-Colasante X. Bender D. E. Yung R.. (2016). Expression of microRNAs in human post-mortem amyotrophic lateral sclerosis spinal cords provides insight into disease mechanisms. Mol. Cell. Neurosci. 71, 34–45. 10.1016/j.mcn.2015.12.008 26704906 PMC4761498

[B30] Figueroa-Romero C. Iñiguez-Lluhí J. A. Stadler J. Chang C. R. Arnoult D. Keller P. J.. (2009). SUMOylation of the mitochondrial fission protein Drp1 occurs at multiple nonconsensus sites within the B domain and is linked to its activity cycle. FASEB J. 23, 3917–3927. 10.1096/fj.09-136630 19638400 PMC2775011

[B31] Freischmidt A. Müller K. Ludolph A. C. Weishaupt J. H. (2013). Systemic dysregulation of TDP-43 binding microRNAs in amyotrophic lateral sclerosis. Acta Neuropathol. Commun. 1:42. 10.1186/2051-5960-1-42 24252274 PMC3893596

[B32] Gagliardi D. Comi G. P. Bresolin N. Corti S. (2019). MicroRNAs as regulators of cell death mechanisms in amyotrophic lateral sclerosis. J. Cell. Mol. Med. 23, 1647–1656. 10.1111/jcmm.13976 30614179 PMC6378226

[B33] Gao J. Wang L. Huntley M. L. Perry G. Wang X. (2018). Pathomechanisms of TDP-43 in neurodegeneration. J. Neurochem. 146, 7–20. 10.1111/jnc.14327 29486049 PMC6110993

[B34] Goutman S. A. Chen K. S. Paez-Colasante X. Feldman E. L. (2018). Emerging understanding of the genotype-phenotype relationship in amyotrophic lateral sclerosis. Handb. Clin. Neurol. 148, 603–623. 10.1016/B978-0-444-64076-5.00039-9 29478603

[B35] Gu J. Chen F. Iqbal K. Gong C. X. Wang X. Liu F. (2017). Transactive response DNA-binding protein 43 (TDP-43) regulates alternative splicing of tau exon 10: Implications for the pathogenesis of tauopathies. J. Biol. Chem. 292, 10600–10612. 10.1074/jbc.M117.783498 28487370 PMC5481566

[B36] Ha M. Kim V. N. (2014). Regulation of microRNA biogenesis. Nat. Rev. Mol. Cell Biol. 15, 509–524. 10.1038/nrm3838 25027649

[B37] Hardiman O. Al-Chalabi A. Chio A. Corr E. M. Logroscino G. Robberecht W.. (2017). Amyotrophic lateral sclerosis. Nat. Rev. Dis. Primers 3:17071. 10.1038/nrdp.2017.71 28980624

[B38] Hollville E. Romero S. E. Deshmukh M. (2019). Apoptotic cell death regulation in neurons. FEBS J. 286, 3276–3298. 10.1111/febs.14970 31230407 PMC6718311

[B39] Honda D. Ishigaki S. Iguchi Y. Fujioka Y. Udagawa T. Masuda A.. (2013). The ALS/FTLD-related RNA-binding proteins TDP-43 and FUS have common downstream RNA targets in cortical neurons. FEBS Open Bio 4, 1–10. 10.1016/j.fob.2013.11.001 24319651 PMC3851184

[B40] Iguchi Y. Katsuno M. Niwa J. Takagi S. Ishigaki S. Ikenaka K.. (2013). Loss of TDP-43 causes age-dependent progressive motor neuron degeneration. Brain 136, 1371–1382. 10.1093/brain/awt029 23449777

[B41] Janssens J. Wils H. Kleinberger G. Joris G. Cuijt I. Ceuterick-de Groote C.. (2013). Overexpression of ALS-associated p.M337V human TDP-43 in mice worsens disease features compared to wild-type human TDP-43 mice. Mol. Neurobiol. 48, 22–35. 10.1007/s12035-013-8427-5 23475610 PMC3718993

[B42] Jimenez-Pacheco A. Franco J. M. Lopez S. Gomez-Zumaquero J. M. Magdalena Leal-Lasarte M. Caballero-Hernandez D. E.. (2017). Epigenetic mechanisms of gene regulation in amyotrophic lateral sclerosis. Adv. Exp. Med. Biol. 978, 255–275. 10.1007/978-3-319-53889-1_14 28523551

[B43] Jobe E. M. McQuate A. L. Zhao X. (2012). Crosstalk among epigenetic pathways regulates neurogenesis. Front. Neurosci. 6:59. 10.3389/fnins.2012.00059 22586361 PMC3347638

[B44] Joilin G. Leigh P. N. Newbury S. F. Hafezparast M. (2019). An overview of MicroRNAs as biomarkers of ALS. Front. Neurol. 10:186. 10.3389/fneur.2019.00186 30899244 PMC6416171

[B45] Kapeli K. Martinez F. J. Yeo G. W. (2017). Genetic mutations in RNA-binding proteins and their roles in ALS. Hum. Genet. 136, 1193–1214. 10.1007/s00439-017-1830-7 28762175 PMC5602095

[B46] Katsu M. Hama Y. Utsumi J. Takashina K. Yasumatsu H. Mori F.. (2019). MicroRNA expression profiles of neuron-derived extracellular vesicles in plasma from patients with amyotrophic lateral sclerosis. Neurosci. Lett. 708:134176. 10.1016/j.neulet.2019.03.048 31173847

[B47] Katsuno M. Adachi H. Banno H. Suzuki K. Tanaka F. Sobue G. (2011). Transforming growth factor-β signaling in motor neuron diseases. Curr. Mol. Med. 11, 48–56. 10.2174/156652411794474356 21189118

[B48] Kawahara Y. Mieda-Sato A. (2012). TDP-43 promotes microRNA biogenesis as a component of the Drosha and Dicer complexes. Proc. Natl. Acad. Sci. U S A 109, 3347–3352. 10.1073/pnas.1112427109 22323604 PMC3295278

[B49] King I. Yartseva V. Salas D. Kumar A. Heidersbach A. Ando D. M.. (2014). The RNA binding protein TDP-43 selectively disrupts MicroRNA-1/206 incorporation into the RNA-induced silencing complex. J. Biol. Chem. 289, 14263–14271. 10.1074/jbc.M114.561902 24719334 PMC4022891

[B50] Kobayashi H. Tomari Y. (2016). RISC assembly: coordination between small RNAs and Argonaute proteins. Biochim. Biophys. Acta 1859, 71–81. 10.1016/j.bbagrm.2015.08.007 26303205

[B51] Kovanda A. Leonardis L. Zidar J. Koritnik B. Dolenc-Groselj L. Ristic Kovacic S.. (2018). Differential expression of microRNAs and other small RNAs in muscle tissue of patients with ALS and healthy age-matched controls. Sci. Rep. 8:5609. 10.1038/s41598-018-23139-2 29618798 PMC5884852

[B52] Kwiatkowski T. J. Jr. Bosco D. A. Leclerc A. L. Tamrazian E. Vanderburg C. R. Russ C.. (2009). Mutations in the FUS/TLS gene on chromosome 16 cause familial amyotrophic lateral sclerosis. Science 323, 1205–1208. 10.1126/science.1166066 19251627

[B53] Lagos-Quintana M. Rauhut R. Lendeckel W. Tuschl T. (2001). Identification of novel genes coding for small expressed RNAs. Science 294, 853–858. 10.1126/science.1064921 11679670

[B54] Lau N. C. Lim L. P. Weinstein E. G. Bartel D. P. (2001). An abundant class of tiny RNAs with probable regulatory roles in Caenorhabditis elegans. Science 294, 858–862. 10.1126/science.1065062 11679671

[B55] Lee R. C. Ambros V. (2001). An extensive class of small RNAs in Caenorhabditis elegans. Science 294, 862–864. 10.1126/science.1065329 11679672

[B57] Li X. Q. Chen F. S. Tan W. F. Fang B. Zhang Z. L. Ma H. (2017). Elevated microRNA-129–5p level ameliorates neuroinflammation and blood-spinal cord barrier damage after ischemia-reperfusion by inhibiting HMGB1 and the TLR3-cytokine pathway. J. Neuroinflammation 14:205. 10.1186/s12974-017-0977-4 29061187 PMC5654055

[B56] Li Q. Yokoshi M. Okada H. Kawahara Y. (2015). The cleavage pattern of TDP-43 determines its rate of clearance and cytotoxicity. Nat. Commun. 6:6183. 10.1038/ncomms7183 25630387

[B58] Liguori M. Nuzziello N. Introna A. Consiglio A. Licciulli F. D’Errico E.. (2018). Dysregulation of MicroRNAs and target genes networks in peripheral blood of patients with sporadic amyotrophic lateral sclerosis. Front. Mol. Neurosci. 11:288. 10.3389/fnmol.2018.00288 30210287 PMC6121079

[B59] Ling S. C. Albuquerque C. P. Han J. S. Lagier-Tourenne C. Tokunaga S. Zhou H.. (2010). ALS-associated mutations in TDP-43 increase its stability and promote TDP-43 complexes with FUS/TLS. Proc. Natl. Acad. Sci. U S A 107, 13318–13323. 10.1073/pnas.1008227107 20624952 PMC2922163

[B60] Liu A. H. Wu Y. T. Wang Y. P. (2017). MicroRNA-129–5p inhibits the development of autoimmune encephalomyelitis-related epilepsy by targeting HMGB1 through the TLR4/NF-κB signaling pathway. Brain Res. Bull. 132, 139–149. 10.1016/j.brainresbull.2017.05.004 28528202

[B61] Liu-Yesucevitz L. Bilgutay A. Zhang Y. J. Vanderweyde T. Citro A. Mehta T.. (2010). Tar DNA binding protein-43 (TDP-43) associates with stress granules: analysis of cultured cells and pathological brain tissue. PLoS One 5:e13250. 10.1371/journal.pone.0013250 20948999 PMC2952586

[B62] Loffreda A. Rigamonti A. Barabino S. M. Lenzken S. C. (2015). RNA-binding proteins in the regulation of miRNA activity: a focus on neuronal functions. Biomolecules 5, 2363–2387. 10.3390/biom5042363 26437437 PMC4693239

[B63] López-González M. J. Soula A. Landry M. Favereaux A. (2018). Oxaliplatin treatment impairs extension of sensory neuron neurites *in vitro* through miR-204 overexpression. Neurotoxicology 68, 91–100. 10.1016/j.neuro.2018.07.009 30031110

[B64] Lunn J. S. Sakowski S. A. Kim B. Rosenberg A. A. Feldman E. L. (2009). Vascular endothelial growth factor prevents G93A-SOD1-induced motor neuron degeneration. Dev. Neurobiol. 69, 871–884. 10.1002/dneu.20747 19672955 PMC2853013

[B65] Maoz R. Garfinkel B. P. Soreq H. (2017). Alzheimer’s disease and ncRNAs. Adv. Exp. Med. Biol. 978, 337–361. 10.1007/978-3-319-53889-1_18 28523555

[B66] M’Boutchou M. N. van Kempen L. C. (2016). Analysis of the tumor microenvironment transcriptome *via* NanoString mRNA and miRNA expression profiling. Methods Mol. Biol. 1458, 291–310. 10.1007/978-1-4939-3801-8_21 27581030

[B67] Meroni M. Crippa V. Cristofani R. Rusmini P. Cicardi M. E. Messi E.. (2019). Transforming growth factor β 1 signaling is altered in the spinal cord and muscle of amyotrophic lateral sclerosis mice and patients. Neurobiol. Aging 82, 48–59. 10.1016/j.neurobiolaging.2019.07.001 31394426

[B68] Miguel L. Frebourg T. Campion D. Lecourtois M. (2011). Both cytoplasmic and nuclear accumulations of the protein are neurotoxic in *Drosophila* models of TDP-43 proteinopathies. Neurobiol. Dis. 41, 398–406. 10.1016/j.nbd.2010.10.007 20951205

[B69] Neumann M. Kwong L. K. Lee E. B. Kremmer E. Flatley A. Xu Y.. (2009). Phosphorylation of S409/410 of TDP-43 is a consistent feature in all sporadic and familial forms of TDP-43 proteinopathies. Acta Neuropathol. 117, 137–149. 10.1007/s00401-008-0477-9 19125255 PMC2693625

[B70] Neumann M. Sampathu D. M. Kwong L. K. Truax A. C. Micsenyi M. C. Chou T. T.. (2006). Ubiquitinated TDP-43 in frontotemporal lobar degeneration and amyotrophic lateral sclerosis. Science 314, 130–133. 10.1126/science.1134108 17023659

[B71] Oskarsson B. Gendron T. F. Staff N. P. (2018). Amyotrophic lateral sclerosis: an update for 2018. Mayo Clin. Proc. 93, 1617–1628. 10.1016/j.mayocp.2018.04.007 30401437

[B72] Paez-Colasante X. Figueroa-Romero C. Sakowski S. A. Goutman S. A. Feldman E. L. (2015). Amyotrophic lateral sclerosis: mechanisms and therapeutics in the epigenomic era. Nat. Rev. Neurol. 11, 266–279. 10.1038/nrneurol.2015.57 25896087

[B73] Pekarsky Y. Balatti V. Croce C. M. (2018). BCL2 and miR-15/16: from gene discovery to treatment. Cell Death Differ. 25, 21–26. 10.1038/cdd.2017.159 28984869 PMC5729525

[B74] Poddar S. Kesharwani D. Datta M. (2017). Interplay between the miRNome and the epigenetic machinery: implications in health and disease. J. Cell Physiol. 232, 2938–2945. 10.1002/jcp.25819 28112397

[B75] Prasad A. Bharathi V. Sivalingam V. Girdhar A. Patel B. K. (2019). Molecular mechanisms of TDP-43 misfolding and pathology in amyotrophic lateral sclerosis. Front. Mol. Neurosci. 12:25. 10.3389/fnmol.2019.00025 30837838 PMC6382748

[B76] Quinlan S. Kenny A. Medina M. Engel T. Jimenez-Mateos E. M. (2017). MicroRNAs in Neurodegenerative Diseases. Int Rev Cell Mol Biol 334, 309–343. 10.1016/bs.ircmb.2017.04.002 28838542

[B77] Rajman M. Metge F. Fiore R. Khudayberdiev S. Aksoy-Aksel A. Bicker S.. (2017). A microRNA-129–5p/Rbfox crosstalk coordinates homeostatic downscaling of excitatory synapses. EMBO J. 36, 1770–1787. 10.15252/embj.201695748 28487411 PMC5470041

[B78] Ramsey J. E. Kelm R. J. Jr. (2009). Mechanism of strand-specific smooth muscle α-actin enhancer interaction by purine-rich element binding protein B (Purβ). Biochemistry 48, 6348–6360. 10.1021/bi900708j 19496623 PMC2752054

[B79] Rinchetti P. Rizzuti M. Faravelli I. Corti S. (2018). MicroRNA metabolism and dysregulation in amyotrophic lateral sclerosis. Mol. Neurobiol. 55, 2617–2630. 10.1007/s12035-017-0537-z 28421535

[B80] Rumora A. E. Steere A. N. Ramsey J. E. Knapp A. M. Ballif B. A. Kelm R. J. Jr. (2010). Isolation and characterization of the core single-stranded DNA-binding domain of purine-rich element binding protein B (Purβ). Biochem. Biophys. Res. Commun. 400, 340–345. 10.1016/j.bbrc.2010.08.059 20728429 PMC2957832

[B81] Rumora A. E. Wang S. X. Ferris L. A. Everse S. J. Kelm R. J. Jr. (2013). Structural basis of multisite single-stranded DNA recognition and ACTA2 repression by purine-rich element binding protein B (Purβ). Biochemistry 52, 4439–4450. 10.1021/bi400283r 23724822 PMC3750979

[B82] Ryan J. A. (2008). Cell Cloning by Serial Dilution in 96 Well Plates Protocol. Corning, NY: Corning Incorporated Available online at: https://www.corning.com/catalog/cls/documents/protocols/Single_cell_cloning_protocol.pdf.

[B83] Selbach M. Schwanhausser B. Thierfelder N. Fang Z. Khanin R. Rajewsky N. (2008). Widespread changes in protein synthesis induced by microRNAs. Nature 455, 58–63. 10.1038/nature07228 18668040

[B84] Sephton C. F. Cenik C. Kucukural A. Dammer E. B. Cenik B. Han Y.. (2011). Identification of neuronal RNA targets of TDP-43-containing ribonucleoprotein complexes. J. Biol. Chem. 286, 1204–1215. 10.1074/jbc.M110.190884 21051541 PMC3020728

[B85] Sephton C. F. Good S. K. Atkin S. Dewey C. M. Mayer P. III. Herz J.. (2010). TDP-43 is a developmentally regulated protein essential for early embryonic development. J. Biol. Chem. 285, 6826–6834. 10.1074/jbc.M109.061846 20040602 PMC2825476

[B86] Sharma S. De Carvalho D. D. Jeong S. Jones P. A. Liang G. (2011). Nucleosomes containing methylated DNA stabilize DNA methyltransferases 3A/3B and ensure faithful epigenetic inheritance. PLoS Genet. 7:e1001286. 10.1371/journal.pgen.1001286 21304883 PMC3033376

[B87] Si Y. Cui X. Crossman D. K. Hao J. Kazamel M. Kwon Y.. (2018). Muscle microRNA signatures as biomarkers of disease progression in amyotrophic lateral sclerosis. Neurobiol. Dis. 114, 85–94. 10.1016/j.nbd.2018.02.009 29486297 PMC5891369

[B88] Si Y. Cui X. Kim S. Wians R. Sorge R. Oh S. J.. (2014). Smads as muscle biomarkers in amyotrophic lateral sclerosis. Ann. Clin. Transl. Neurol. 1, 778–787. 10.1002/acn3.117 25493269 PMC4241805

[B89] Si Y. Kim S. Cui X. Zheng L. Oh S. J. Anderson T.. (2015). Transforming growth factor β (TGF-β) is a muscle biomarker of disease progression in ALS and correlates with smad expression. PLoS One 10:e0138425. 10.1371/journal.pone.0138425 26375954 PMC4574401

[B90] Singh A. Sen D. (2017). MicroRNAs in Parkinson’s disease. Exp. Brain Res. 235, 2359–2374. 10.1007/s00221-017-4989-1 28526930

[B91] Soane L. Fiskum G. (2005). Inhibition of mitochondrial neural cell death pathways by protein transduction of Bcl-2 family proteins. J. Bioenerg. Biomembr. 37, 179–190. 10.1007/s10863-005-6590-8 16167175 PMC2570496

[B92] Sosanya N. M. Brager D. H. Wolfe S. Niere F. Raab-Graham K. F. (2015). Rapamycin reveals an mTOR-independent repression of Kv1.1 expression during epileptogenesis. Neurobiol. Dis. 73, 96–105. 10.1016/j.nbd.2014.09.011 25270294

[B93] Spiller K. J. Cheung C. J. Restrepo C. R. Kwong L. K. Stieber A. M. Trojanowski J. Q.. (2016a). Selective motor neuron resistance and recovery in a new inducible mouse model of TDP-43 proteinopathy. J. Neurosci. 36, 7707–7717. 10.1523/jneurosci.1457-16.2016 27445147 PMC6705561

[B96] Spiller K. J. Restrepo C. R. Khan T. Stieber A. M. Kwong L. K. Trojanowski J. Q.. (2016b). Progression of motor neuron disease is accelerated and the ability to recover is compromised with advanced age in rNLS8 mice. Acta Neuropathol. Commun. 4:105. 10.1186/s40478-016-0377-5 27687289 PMC5043606

[B94] Spiller K. J. Khan T. Dominique M. A. Restrepo C. R. Cotton-Samuel D. Levitan M.. (2019). Reduction of matrix metalloproteinase 9 (MMP-9) protects motor neurons from TDP-43-triggered death in rNLS8 mice. Neurobiol. Dis. 124, 133–140. 10.1016/j.nbd.2018.11.013 30458231 PMC7053168

[B95] Spiller K. J. Restrepo C. R. Khan T. Dominique M. A. Fang T. C. Canter R. G.. (2018). Microglia-mediated recovery from ALS-relevant motor neuron degeneration in a mouse model of TDP-43 proteinopathy. Nat. Neurosci. 21, 329–340. 10.1038/s41593-018-0083-7 29463850 PMC5857237

[B97] Sreedharan J. Blair I. P. Tripathi V. B. Hu X. Vance C. Rogelj B.. (2008). TDP-43 mutations in familial and sporadic amyotrophic lateral sclerosis. Science 319, 1668–1672. 10.1126/science.1154584 18309045 PMC7116650

[B98] Taguchi Y. H. Wang H. (2018). Exploring microRNA biomarker for amyotrophic lateral sclerosis. Int. J. Mol. Sci. 19:E1318. 10.3390/ijms19051318 29710810 PMC5983737

[B99] Takahashi I. Hama Y. Matsushima M. Hirotani M. Kano T. Hohzen H.. (2015). Identification of plasma microRNAs as a biomarker of sporadic Amyotrophic Lateral Sclerosis. Mol. Brain 8:67. 10.1186/s13041-015-0161-7 26497046 PMC4619470

[B100] Talepoor Ardakani M. Rostamian Delavar M. Baghi M. Nasr-Esfahani M. H. Kiani-Esfahani A. Ghaedi K. (2019). Upregulation of miR-200a and miR-204 in MPP^+^ -treated differentiated PC12 cells as a model of Parkinson’s disease. Mol. Genet. Genomic Med. 7:e548. 10.1002/mgg3.548 30712312 PMC6418372

[B101] Tan L. Yu J. T. Tan L. (2015). Causes and consequences of MicroRNA dysregulation in neurodegenerative diseases. Mol. Neurobiol. 51, 1249–1262. 10.1007/s12035-014-8803-9 24973986

[B102] Vance C. Rogelj B. Hortobagyi T. De Vos K. J. Nishimura A. L. Sreedharan J.. (2009). Mutations in FUS, an RNA processing protein, cause familial amyotrophic lateral sclerosis type 6. Science 323, 1208–1211. 10.1126/science.1165942 19251628 PMC4516382

[B103] Vlachos I. S. Zagganas K. Paraskevopoulou M. D. Georgakilas G. Karagkouni D. Vergoulis T.. (2015). DIANA-miRPath v3.0: deciphering microRNA function with experimental support. Nucleic Acids Res. 43, W460–W466. 10.1093/nar/gkv403 25977294 PMC4489228

[B104] Vucic S. Rothstein J. D. Kiernan M. C. (2014). Advances in treating amyotrophic lateral sclerosis: insights from pathophysiological studies. Trends Neurosci. 37, 433–442. 10.1016/j.tins.2014.05.006 24927875

[B105] Walker A. K. Spiller K. J. Ge G. Zheng A. Xu Y. Zhou M.. (2015). Functional recovery in new mouse models of ALS/FTLD after clearance of pathological cytoplasmic TDP-43. Acta Neuropathol. 130, 643–660. 10.1007/s00401-015-1460-x 26197969 PMC5127391

[B106] Wang N. Yang W. Xiao T. Miao Z. Luo W. You Z.. (2018). Possible role of miR-204 in optic nerve injury through the regulation of GAP-43. Mol. Med. Rep. 17, 3891–3897. 10.3892/mmr.2017.8341 29286154

[B107] Wang X. Zhou S. Ding X. Ma M. Zhang J. Zhou Y.. (2015). Activation of ER stress and autophagy induced by TDP-43 A315T as pathogenic mechanism and the corresponding histological changes in skin as potential biomarker for ALS with the mutation. Int. J. Biol. Sci. 11, 1140–1149. 10.7150/ijbs.12657 26327808 PMC4551750

[B108] Wegorzewska I. Baloh R. H. (2011). TDP-43-based animal models of neurodegeneration: new insights into ALS pathology and pathophysiology. Neurodegener. Dis. 8, 262–274. 10.1159/000321547 21124004 PMC3214943

[B109] Weskamp K. Barmada S. J. (2018). TDP43 and RNA instability in amyotrophic lateral sclerosis. Brain Res. 1693, 67–74. 10.1016/j.brainres.2018.01.015 29395044 PMC5997512

[B110] Weskamp K. Tank E. M. Miguez R. McBride J. P. Gomez N. B. White M.. (2020). Shortened TDP43 isoforms upregulated by neuronal hyperactivity drive TDP43 pathology in ALS. J. Clin. Invest. 130, 1139–1155. 10.1172/JCI130988 31714900 PMC7269575

[B111] Wils H. Kleinberger G. Janssens J. Pereson S. Joris G. Cuijt I.. (2010). TDP-43 transgenic mice develop spastic paralysis and neuronal inclusions characteristic of ALS and frontotemporal lobar degeneration. Proc. Natl. Acad. Sci. U S A 107, 3858–3863. 10.1073/pnas.0912417107 20133711 PMC2840518

[B112] Winton M. J. Igaz L. M. Wong M. M. Kwong L. K. Trojanowski J. Q. Lee V. M. (2008). Disturbance of nuclear and cytoplasmic TAR DNA-binding protein (TDP-43) induces disease-like redistribution, sequestration, and aggregate formation. J. Biol. Chem. 283, 13302–13309. 10.1074/jbc.m800342200 18305110 PMC2442318

[B113] Wohlfarth C. Schmitteckert S. Hartle J. D. Houghton L. A. Dweep H. Fortea M. (2017). miR-16 and miR-103 impact 5-HT4 receptor signalling and correlate with symptom profile in irritable bowel syndrome. Sci. Rep. 7:14680 10.1038/s41598-017-13982-0 29089619 PMC5665867

[B114] Yan L. Shi E. Jiang X. Shi J. Gao S. Liu H. (2019). Inhibition of MicroRNA-204 conducts neuroprotection against spinal cord ischemia. Ann. Thorac. Surg. 107, 76–83. 10.1016/j.athoracsur.2018.07.082 30278168

[B115] Zeng Z. Liu Y. Zheng W. Liu L. Yin H. Zhang S.. (2019). MicroRNA-129–5p alleviates nerve injury and inflammatory response of Alzheimer’s disease *via* downregulating SOX6. Cell Cycle 18, 3095–3110. 10.1080/15384101.2019.1669388 31564203 PMC6816367

[B116] Zhang H. Tan C. F. Mori F. Tanji K. Kakita A. Takahashi H.. (2008). TDP-43-immunoreactive neuronal and glial inclusions in the neostriatum in amyotrophic lateral sclerosis with and without dementia. Acta Neuropathol. 115, 115–122. 10.1007/s00401-007-0285-7 17786458

