# Supplementary material for: Cytoplasmic TDP43 Binds microRNAs: New Disease Targets in Amyotrophic Lateral Sclerosis
Source: Front Cell Neurosci. 2020 May 12;14:117. doi: 10.3389/fncel.2020.00117 (PMC7235295; doi:10.3389/fncel.2020.00117)
Supplement: Supplementary file 1 [file Table_1.DOCX]

| **Supplementary Table 1. Predicted pathways represented among the ΔNLS-TDP43 enriched miRNAs (n=52)** | | | |
| --- | --- | --- | --- |
| **KEGG pathway** | **p-value** | **#genes** | **#miRNAs** |
| ECM-receptor interaction | 6.70E-08 | 47 | 36 |
| Hippo signaling pathway | 1.53E-07 | 99 | 42 |
| Pathways in cancer | 4.95E-07 | 243 | 48 |
| Fatty acid biosynthesis | 7.06E-07 | 8 | 17 |
| TGF-beta signaling pathway | 7.06E-07 | 55 | 37 |
| Signaling pathways regulating pluripotency of stem cells | 3.98E-06 | 93 | 43 |
| FoxO signaling pathway | 1.34E-05 | 91 | 39 |
| ErbB signaling pathway | 2.77E-05 | 63 | 40 |
| Adrenergic signaling in cardiomyocytes | 5.74E-05 | 93 | 42 |
| Renal cell carcinoma | 0.000107141 | 47 | 37 |
| Morphine addiction | 0.000107141 | 59 | 38 |
| Proteoglycans in cancer | 0.000107141 | 121 | 41 |
| Lysine degradation | 0.000163457 | 31 | 32 |
| Glioma | 0.000163457 | 44 | 37 |
| GABAergic synapse | 0.000163457 | 54 | 38 |
| Axon guidance | 0.000163457 | 83 | 41 |
| Wnt signaling pathway | 0.00016376 | 90 | 44 |
| Focal adhesion | 0.000295871 | 129 | 40 |
| Melanogenesis | 0.000390693 | 67 | 41 |
| Rap1 signaling pathway | 0.000390693 | 130 | 44 |
| MAPK signaling pathway | 0.000532291 | 155 | 47 |
| Glutamatergic synapse | 0.000583187 | 70 | 41 |
| Retrograde endocannabinoid signaling | 0.000752707 | 66 | 40 |
| Prostate cancer | 0.000843156 | 60 | 39 |
| Ras signaling pathway | 0.000863633 | 133 | 43 |
| Estrogen signaling pathway | 0.001231848 | 60 | 40 |
| Mucin type O-Glycan biosynthesis | 0.00262994 | 17 | 20 |
| Chronic myeloid leukemia | 0.003128637 | 50 | 37 |
| mTOR signaling pathway | 0.003261009 | 43 | 34 |
| PI3K-Akt signaling pathway | 0.003596696 | 194 | 45 |
| N-Glycan biosynthesis | 0.003710123 | 27 | 27 |
| Glycosaminoglycan biosynthesis - heparan sulfate / heparin | 0.00396943 | 17 | 20 |
| Prion diseases | 0.00396943 | 13 | 21 |
| Melanoma | 0.004724066 | 49 | 36 |
| mRNA surveillance pathway | 0.012225822 | 57 | 36 |
| p53 signaling pathway | 0.012646318 | 45 | 33 |
| Dopaminergic synapse | 0.012847181 | 80 | 43 |
| Transcriptional misregulation in cancer | 0.013178554 | 99 | 40 |
| Choline metabolism in cancer | 0.013783845 | 66 | 39 |
| Small cell lung cancer | 0.025902308 | 54 | 35 |
| Gap junction | 0.027321655 | 54 | 38 |
| Nicotine addiction | 0.040187853 | 24 | 32 |
| Pancreatic cancer | 0.040845619 | 42 | 34 |
| Neurotrophin signaling pathway | 0.040926583 | 74 | 41 |
| Adherens junction | 0.042366264 | 51 | 36 |
| *Shading indicates pathways altered in both cell lines* | | | |

**Supplementary Material**
