# Supplementary material for: Cytoplasmic TDP43 Binds microRNAs: New Disease Targets in Amyotrophic Lateral Sclerosis
Source: Front Cell Neurosci. 2020 May 12;14:117. doi: 10.3389/fncel.2020.00117 (PMC7235295; doi:10.3389/fncel.2020.00117)
Supplement: Supplementary file 2 [file Table_2.DOCX]

| **Supplementary Table 2. Predicted pathways represented among the WT-TDP43 enriched miRNAs (n=24)** | | | |
| --- | --- | --- | --- |
| **KEGG pathway** | **p-value** | **#genes** | **#miRNAs** |
| Fatty acid biosynthesis | 1.12E-09 | 7 | 9 |
| Proteoglycans in cancer | 1.15E-09 | 109 | 23 |
| Signaling pathways regulating pluripotency of stem cells | 2.44E-07 | 78 | 23 |
| Wnt signaling pathway | 7.44E-07 | 79 | 23 |
| TGF-beta signaling pathway | 1.61E-06 | 45 | 21 |
| Melanogenesis | 1.61E-06 | 61 | 23 |
| Prion diseases | 1.34E-05 | 12 | 14 |
| Lysine degradation | 6.71E-05 | 26 | 19 |
| Adrenergic signaling in cardiomyocytes | 0.000136733 | 75 | 23 |
| Hippo signaling pathway | 0.000174349 | 81 | 23 |
| Axon guidance | 0.000186197 | 68 | 20 |
| Morphine addiction | 0.000353656 | 47 | 21 |
| Pathways in cancer | 0.000353656 | 187 | 23 |
| Endocrine and other factor-regulated calcium reabsorption | 0.000370753 | 25 | 19 |
| Glutamatergic synapse | 0.00040103 | 59 | 21 |
| ErbB signaling pathway | 0.00057494 | 52 | 23 |
| Rap1 signaling pathway | 0.00061741 | 106 | 24 |
| Estrogen signaling pathway | 0.000832107 | 49 | 22 |
| Glioma | 0.001258677 | 35 | 21 |
| Adherens junction | 0.001363224 | 44 | 19 |
| FoxO signaling pathway | 0.00138805 | 69 | 21 |
| Renal cell carcinoma | 0.001536942 | 39 | 21 |
| GABAergic synapse | 0.001609039 | 43 | 20 |
| Prostate cancer | 0.001791377 | 49 | 23 |
| mTOR signaling pathway | 0.001795883 | 37 | 20 |
| Retrograde endocannabinoid signaling | 0.00236326 | 52 | 21 |
| Ubiquitin mediated proteolysis | 0.002396717 | 73 | 21 |
| Gap junction | 0.009533856 | 42 | 21 |
| Oxytocin signaling pathway | 0.010546071 | 79 | 23 |
| Dopaminergic synapse | 0.010772117 | 67 | 23 |
| HTLV-I infection | 0.011630862 | 121 | 24 |
| cGMP-PKG signaling pathway | 0.014643481 | 80 | 23 |
| cAMP signaling pathway | 0.015042354 | 94 | 24 |
| Colorectal cancer | 0.018017609 | 35 | 19 |
| Long-term depression | 0.020363348 | 32 | 19 |
| Gastric acid secretion | 0.020363348 | 40 | 23 |
| Long-term potentiation | 0.021102204 | 37 | 22 |
| Cholinergic synapse | 0.024109204 | 58 | 22 |
| Focal adhesion | 0.024454173 | 96 | 23 |
| Neurotrophin signaling pathway | 0.024867506 | 61 | 23 |
| Melanoma | 0.025760052 | 38 | 19 |
| Regulation of actin cytoskeleton | 0.027442021 | 100 | 21 |
| Amphetamine addiction | 0.027442021 | 33 | 21 |
| Oocyte meiosis | 0.027500726 | 58 | 22 |
| Sphingolipid signaling pathway | 0.034385726 | 57 | 20 |
| Ras signaling pathway | 0.034385726 | 100 | 22 |
| PI3K-Akt signaling pathway | 0.034385726 | 147 | 24 |
| Prolactin signaling pathway | 0.048988319 | 35 | 21 |
| *Shading indicates pathways altered in both cell lines* | | | |
